# Efficient and Stable MA‐Free Sn─Pb Perovskite Solar Cells With Exceptional *V*_OC_ by Bioavailable Phytophenol Sinapic Acid

**DOI:** 10.1002/advs.76007

**Published:** 2026-09-01

**Authors:** Seojun Lee, Padmini Pandey, Dong Gyu Lee, Tae‐Gyeong Lee, Saemon Yoon, Insub Noh, Sang Wook Park, Jun Ryu, SungWon Cho, Dong‐Gun Lee, Hyung Do Kim, Tae Kyung Lee, Dong‐Won Kang

**Affiliations:** ^1^ Department of Energy Systems Engineering Chung‐Ang University Seoul Republic of Korea; ^2^ Department of Organic and Nano Engineering Hanyang University Seoul Republic of Korea; ^3^ Human‐Tech Convergence Program Hanyang University Seoul Republic of Korea; ^4^ Department of Polymer Chemistry Graduate School of Engineering Kyoto University Kyoto Japan; ^5^ Department of Materials Engineering and Convergence Technology Gyeongsang National University (GNU) Jinju Republic of Korea; ^6^ Department of Smart Cities Chung‐Ang University Seoul Republic of Korea; ^7^ Department of Materials Science and Engineering Ajou University Suwon Republic of Korea

**Keywords:** methylammonium (MA)‐free, perovskite solar cells (PSCs), sinapic acid, Sn─Pb perovskite, stability, tandem photovoltaics

## Abstract

Narrow‐bandgap Sn─Pb perovskite solar cells (PSCs) are vital for advancing high‐efficiency tandem photovoltaics and near‐infrared optoelectronic devices, yet their performance is limited by Sn^2+^ oxidation, defective crystallization, and instability, particularly in thermally stable methlyammonium (MA)‐free systems. We introduce a transformative approach using sinapic acid (SA), a bioavailable phytophenol, as a multifunctional additive to address these challenges in MA‐free Sn─Pb PSCs. SA forms Lewis acid‐base adducts with Sn^2+^/Pb^2+^ ions, slowing crystallization kinetics to produce compact, uniform films with minimal grain boundary defects. Its electron‐rich functional groups selectively coordinate with uncoordinated metal ions, effectively suppressing Sn^2+^ oxidation and passivating deep trap states that cause non‐radiative recombination. This dual action significantly enhances optoelectronic quality and energy‐level alignment, reducing open‐circuit voltage (*V*_OC_) losses. As a result, the MA‐free Sn─Pb PSC achieves a champion power conversion efficiency (PCE) of 23.2% with a remarkably high *V*_OC_ of 0.893 V, ranking among the highest reported PCEs for Sn─Pb PSCs. In addition, the device exhibits impressive long‐term stability, retaining over 90% of its initial PCE after 5200 h. These findings represent a pivotal advancement in Sn─Pb PSCs development, offering new prospects for highly efficient tandem photovoltaics and narrow‐bandgap perovskites for near‐infrared applications.

## Graphical abstract

Sinapic acid, a bio‐derived phytophenol, serves as a multifunctional additive for methylammonium (MA)‐free Sn─Pb perovskites, coordinating with Sn^2+^/Pb^2+^ to regulate crystallization and mitigate defects. Its electron‐rich groups suppress Sn oxidation and passivate deep traps, improving band alignment and reducing *V*_OC_ losses. Resulting devices achieve a PCE of 23.2%, *V*_OC_ of 0.893 V, and over 90% efficiency retention after 5200 h.

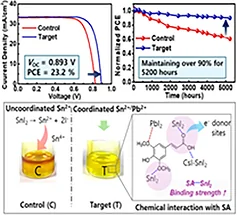

## 1 Introduction

Metal‐halide perovskites have emerged as a transformative platform for photovoltaic (PV) technologies, owing to their solution‐processable nature, low production costs [1], tunable bandgaps [2], superior optoelectronic properties [3], long carrier diffusion lengths [4], low exciton binding energies [5], and high absorption coefficients [6]. The Shockley–Queisser (SQ) limit sets a theoretical efficiency ceiling of approximately 33% for single‐junction (SJ) solar cells with a bandgap (*E*_g_) of 1.34 eV [7]. Perovskite solar cells (PSCs) have achieved remarkable progress, advancing from an initial power conversion efficiency (PCE) of 3.75% to over 27% in a short timeframe [8]. However, conventional lead (Pb)‐based PSCs face challenges, including toxicity concerns [9, 10, 11] and bandgaps often exceeding the ideal 1.1–1.4 eV range, limiting their spectral absorption [7, 12].

To address these limitations, Sn─Pb narrow‐bandgap (NBG) PSCs, achieved by partially substituting Pb^2+^ with Sn^2+^, offer an optimized *E*_g_ of ∼1.25 eV [13, 14]. Theoretical SQ limits suggest Sn─Pb PSCs could exceed 32% PCE, with short‐circuit current density (*J*_SC_) above 39 mA cm^−2^ and an open‐circuit voltage (*V*_OC_) over 0.9 V [15]. However, Sn^2+^’s high reactivity leads to uncontrolled crystallization, poor film morphology, and mobile ionic defects, impairing charge extraction and causing significant *V*_OC_ losses. Most Sn─Pb PSCs rely on methylammonium (MA) cations [16, 17, 18], but MA's volatility accelerates degradation, compromising long‐term stability [19, 20]. MA‐free Sn─Pb PSCs, while promising for enhanced stability, often underperform due to persistent Sn^2+^ oxidation, which creates deep defects and exacerbates non‐radiative recombination.

This study presents a breakthrough in developing efficient, stable MA‐free Sn─Pb PSCs by incorporating *n*‐butylammonium (*n*‐BA), formamidinium (FA), cesium (Cs) cations, and Al‐NiO_x_ (ANO) nanocrystals for hole transport layer (HTL), as reported in our prior work [21, 22]. To overcome Sn^2+^ oxidation and defect challenges, we introduce sinapic acid (SA), a bioavailable phytophenol, as a multifunctional additive. SA's methoxy (CH_3_O─), carboxyl (COO─), and hydroxy (─OH) groups, combined with its conjugated phenyl ring, enable multiple roles: (a) forming Lewis acid‐base adducts with perovskite precursors to regulate crystallization kinetics, (b) ensuring uniform, defect‐free films, and (c) selectively passivating uncoordinated Sn^2+^/Pb^2+^ ions to suppress oxidation and trap states. SA is a naturally occurring hydroxycinnamic acid that is accessible from biomass‐derived sources, making it attractive from the standpoint of practical accessibility and potentially more sustainable additive design [23, 24]. These mechanisms minimize non‐radiative recombination and optimize optoelectronic properties. Consequently, our MA‐free Sn─Pb PSCs achieve an unprecedented PCE of 23.2% with an exceptional *V*_OC_ of 0.893 V, among the highest reported for MA‐free Sn─Pb PSCs. Moreover, devices exhibit unparalleled stability, retaining over 90% of their initial PCE after 5200 h. This work marks a significant advancement toward scalable, eco‐friendly Sn─Pb PSCs for sustainable energy applications.

## 2 Results and Discussion

### 2.1 Lewis Acid‐Base Adduct and Chemical Coordination Analysis

Sinapic acid (SA), a bioavailable phytophenol, features methoxy (CH_3_O─), carboxyl (COO─), hydroxy (─OH), and a conjugated phenyl ring, enabling multifaceted interactions with perovskite precursors (Figure 1A). Density functional theory (DFT) calculations reveal electron‐rich regions in SA, particularly around its functional groups, as shown in the electrostatic potential isosurface (Figure 1A and Figure S1). Moreover, we investigated the role of SA in perovskite crystallization. Firstly, the binding energies were calculated between the A‐site (FA or Cs) cation and BX_3_ (SnI_3_ or PbI_3_) anion for three different systems (i.e., precursors, precursors∙DMSO, and precursors∙DMSO∙SA) to comprehend the binding interactions. Our results show that, in all cases, SA strengthened the interatomic interactions between A‐site cation and BX_3_ anion, potentially affecting the crystallization process by altering precursor binding characteristics (Figure 1B and Figure S2). Interestingly, this effect was the most prominent in the Cs∙SnI_3_ system, which exhibited the greatest increase in negative charge on iodine atoms upon SA molecule addition (39.32%), significantly surpassing the changes observed in Cs∙PbI_3_ and FA‐based systems (Figure S3). The pronounced enhancement in interaction strength, coupled with the considerably increased negative charge on the iodine atoms, strongly suggests that the SA actively promotes the electron accumulation on the iodine sites within the BX_3_ anions. It indicates that the interaction of precursors can be further strengthened, which can potentially improve the perovskite crystallization. This selective enhancement can be rationalized by both structural and electronic factors. First, unlike the FA cation, the Cs cation does not have hydrogen bonding interactions with iodide, rendering the iodine atoms more responsive to changes in the local coordination environment induced by the SA [25]. Second, Sn^2+^ possesses higher Lewis acidity compared to Pb^2+^, and it enables stronger coordination with electron‐donating groups (─OH and COO─) in the SA. These characteristics collectively contribute to the clear observable increase in iodine charge and precursor binding strength in the Cs∙SnI_3_ system.

**FIGURE 1:**
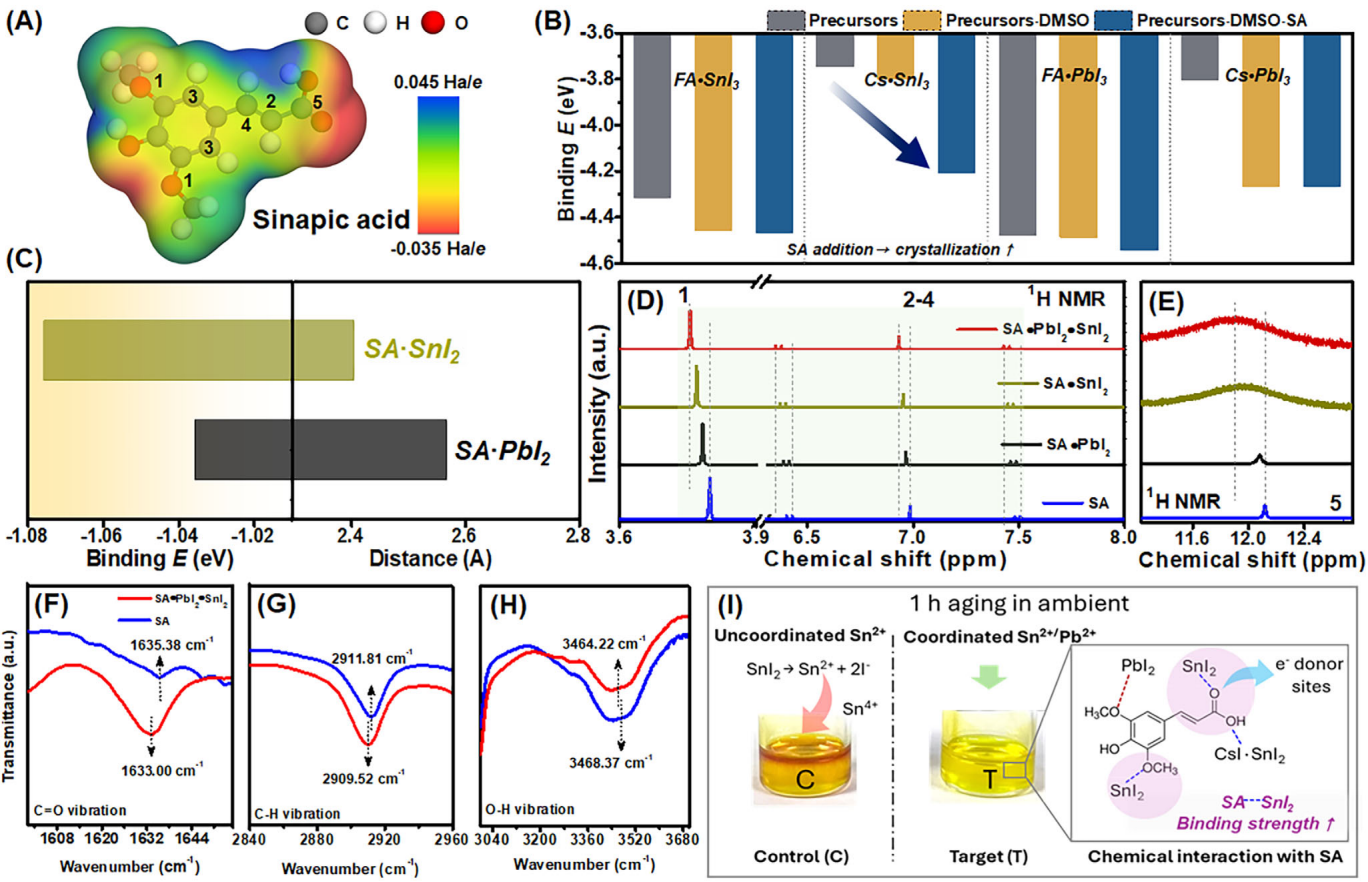
(A) Electrostatic potential isosurface of sinapic acid (SA). The isovalue is 0.03 *e*/Å^3^. (B) Binding energies between precursors under different conditions. Note that precursors indicate that FA·SnI_3_, Cs·SnI_3_, FA·PbI_3_, and Cs·PbI_3_ molecules. (C) Binding energies of SA·SnI_2_ and SA·PbI_2_ and distances from the oxygen atom in SA to the Pb or Sn atom. (D,E) Comparative liquid state ^1^H NMR of selected positions of functionalities in (SA), SA with PbI_2_ (SA∙PbI_2_), SA with SnI_2_ (SA∙SnI_2_), and SA with SnI_2_ and PbI_2_ (SA∙PbI_2_∙SnI_2_) complex. (F–H) Comparative FT‐IR spectral analysis of selected positions of functionalities in SA and its chemical interaction with SnI_2_ and PbI_2_ i.e., SA, and SA∙SnI_2_∙ PbI_2_ complex. (I) Schematic representation revealing the chemical coordination of SA with perovskite precursor solutions.

To probe direct coordination, we compared SA's interactions with SnI_2_ and PbI_2_. DFT results show a lower binding energy for the SA·SnI_2_ complex compared to SA·PbI_2_, indicating stronger, more stable coordination with Sn^2+^ (Figure 1C and Figure S4). The shorter oxygen–Sn distance in SA·SnI_2_ further confirms SA's preferential binding to Sn^2+^, attributed to Sn^2+^’s stronger Lewis acidity due to its reactive 5s electrons. Uncontrolled Sn^2+^ reactivity typically causes rapid nucleation, poor film morphology, and oxidation to Sn^4+^, exacerbating defect formation. SA mitigates these issues by forming stable Lewis acid‐base adducts.

Liquid‐state ^1^H NMR spectroscopy in DMSO‐d_6_ solution corroborated these findings. The SA spectrum matches simulated patterns (Figure S5), with characteristic peaks at δ 12.12 (s, 1H), 7.50 (d, J = 15.9 Hz, 1H), 6.98 (s, 1H), 6.42 (d, J = 15.9 Hz, 1H), and 3.80 (s, 1H) (Figure 1D–E, blue line). Upon coordination with PbI_2_ (SA∙PbI_2_ complex), noticeable chemical shifts were observed: δ 12.09 (s, ^1^H), 8.87 (s, ^1^H), 7.48 (d, J = 15.8 Hz, ^1^H), 6.96 (s, ^1^H), 6.40 (d, J = 15.9 Hz, ^1^H), 3.78 (s, ^1^H) (Figure 1D–E, black line). A more prominent shift occurred in SA∙SnI_2_ complex with peak positions at δ 11.98 (s, ^1^H), 7.47 (d, J = 15.8 Hz, ^1^H), 6.95 (s, ^1^H), 6.39 (d, J = 15.8 Hz, ^1^H), 3.77 (s, ^1^H) (Figure 1D–E, dark yellow line), indicating stronger chemical coordination between SA and SnI_2_ due to faster reaction behavior. Upon introducing both SnI_2_ and PbI_2_ perovskite precursors with SA (SA∙PbI_2_∙SnI_2_ complex), prominent peak shifts were observed at δ 11.89 (s, ^1^H), δ 7.45 (d, J = 15.8 Hz, ^1^H), 6.93 (s, ^1^H), 6.37 (d, J = 15.9 Hz, ^1^H), 3.75 (s, ^1^H) as shown in Figure 1D,E (red line). This indicates that the SA molecule coordinates with both SnI_2_ and PbI_2_, forming a Lewis acid‐base adduct, which is beneficial for crystal growth regulation and crystallinity improvement. In addition, the antioxidant ability of SA, derived from its ‐OH group, allows it to donate electrons to inhibit Sn^2+^/Pb^2+^ oxidation, thereby reducing defect formation. Fourier‐transform infrared (FT‐IR) spectroscopy further validates these interactions. SA's characteristic peaks at 1635.38 cm^−1^ (C═O), 2911.81 cm^−1^ (C─H, phenyl ring), and 3468.37 cm^−1^ (─OH) shift to lower wavenumbers in the SA∙PbI_2_∙SnI_2_ complex, indicating robust adduct formation (Figure 1F–H).

To assess solution stability, we compared precursor solutions (SnI_2_, PbI_2_, CsI, BAI, FAI in DMF:DMSO) with and without SA. The control solution turned dark yellow, signaling Sn^2+^ oxidation and defect formation (Figure 1I and Figure S6). In contrast, the SA‐containing solution remained stable, with no color change, due to SA's multidentate groups (COO─, CH_3_O─, ─OH) forming Sn/Pb···OOC and Sn/Pb···OCH_3_ adducts. These interactions passivate undercoordinated Sn^2+^/Pb^2+^ ions, suppressing trap states and enhancing precursor stability. SA's antioxidant ─OH group further inhibits Sn^2+^ oxidation, reducing defects. Collectively, these results demonstrate SA's critical role in stabilizing Sn─Pb perovskite precursors, regulating crystallization, and enabling high‐quality films for efficient, stable solar cells.

### 2.2 Crystal Growth Analysis and Defect Passivation in Sn─Pb Perovskite Through Sinapic Acid

Field‐emission scanning electron microscopy (FE‐SEM) images further elucidate SA's role in film morphology (Figure 2A–D). Control films exhibit noticeable discontinuities and pinholes on their surface. These morphological defects can facilitate direct contact between the electron transport layer (ETL) and hole transport layer (HTL), leading to increased recombination losses in photovoltaic devices (Figure 2A). In contrast, SA‐treated films show a compact, pinhole‐free morphology (Figure 2B), attributed to defect passivation at grain boundaries and improved crystallinity. Cross‐sectional images corroborate these findings: control films contain voids (Figure 2C), while SA‐treated films are uniform and void‐free (Figure 2D). The Thermogravimetric analysis (TGA) result confirms that SA remains thermally stable during perovskite processing. The decomposition of pure SA begins above ∼200°C (Figure S7), whereas our film annealing temperature is 100°C. Therefore, SA is not expected to decompose during film formation, supporting the view that SA remains chemically intact in the perovskite matrix. To further elucidate the origin of the SA‐induced enhancement of crystallinity, we performed time‐of‐flight secondary ion mass spectrometry (ToF‐SIMS) depth profiling of ITO/HTL/Sn─Pb perovskite films containing SA (Figure S8). In the ToF‐SIMS depth‐profile analysis, the CH_3_O^−^ ion fragment was monitored as a characteristic marker of SA because methoxy groups are an intrinsic part of the intact SA molecular structure. The CH_3_O^−^ signal is detected throughout the perovskite film, with pronounced enrichment near both the film surface and the buried perovskite/HTL (Al─NiO_x_) interface. This distribution indicates that SA is incorporated into the perovskite layer and preferentially accumulates at energetically favorable or defect‐rich regions, particularly at the surface and interfaces.

**FIGURE 2:**
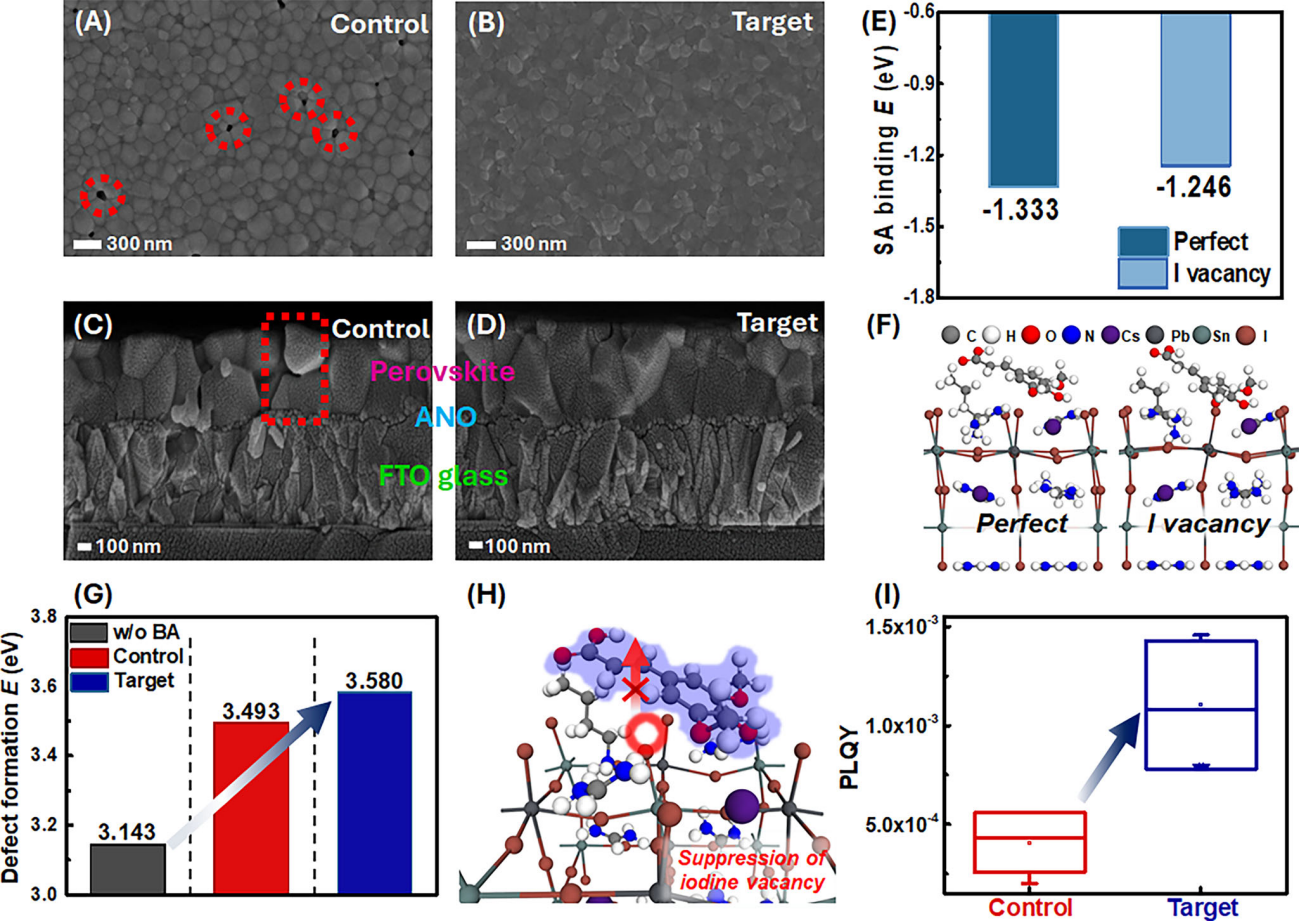
Morphology images of perovskite films of (A) control and (B) target conditions. Cross‐sectional pictures of FTO/Al‐NiO_x_ (ANO) nanocrystals /perovskite films of (C) control and (D) target conditions. (E) Binding energies of the SA molecule on the BAI passivated perovskite surface according to the existence of I vacancy defect. (F) Optimized structures of SA molecule on the perfect and I vacancy systems. (G) Defect formation energies of I atom on the perovskite surface. (H) Schematic representation of the suppression of iodine vacancy by SA in the target system. (I) Photoluminescence quantum yield (PLQY) values of glass/perovskite films for control and target conditions.

Such behavior is likely driven by the strong affinity of SA's functional groups toward undercoordinated Sn^2+^ and Pb^2+^ ions, and the dynamic crystallization environment, which promotes the incorporation or preferential interaction of larger organic molecules like SA at internal interfaces or grain boundaries [17]. Importantly, this preferential localization of SA at the buried interface appears to actively influence the crystallization process. Consistent with this, XRD analysis (Figure S9) showed a pronounced increase in the intensity and sharpening of the (100) diffraction peak in SA‐treated films, while cross‐sectional FE‐SEM images (Figure 2C,D) revealed a denser, more uniform, and vertically oriented grain structure with minimal voids, particularly in the lower perovskite regions, compared to control films. To further clarify the crystallization‐regulating role of SA, annealing‐stage‐resolved ex situ XRD and FE‐SEM measurements were performed for the control and target films under the actual processing sequence (no annealing, 60°C for 1 min, and 100°C for 2, 5, and 10 min; Figures S10–S12). In the no‐annealing state, the control film already showed relatively strong perovskite diffraction, whereas the SA‐treated film exhibited weaker diffraction (Figure S10). Together with the corresponding FE‐SEM images (Figures S11 and S12), this indicates that the control undergoes premature and heterogeneous crystallization before thermal annealing, resulting in severe aggregation, discontinuities, and voids, while SA suppresses this early conversion and shifts crystallization into a more controlled annealing‐driven process, leading to a denser and more compact surface morphology. Consistent with this interpretation, the FE‐SEM images of the final films annealed at 100°C for 10 min revealed a larger average grain size for the SA‐treated film (223 ± 71 nm) than for the control (165 ± 64 nm), further supporting the XRD‐based trend of enhanced final crystallinity with SA.

To explore defect passivation, density functional theory (DFT) calculations assessed SA's binding on perfect and iodine‐vacancy perovskite surfaces (Figures S13 and S14). Confirming this behavior of SA requires that it interacts thermodynamically favorably with the perovskite surface. Therefore, we calculated binding energies of SA on the perovskite surface in perfect and I vacancy systems. (Figure 2E,F and Figure S15). The results indicate that SA exhibits thermodynamically favorable binding on both systems. Importantly, the analysis of the defect formation energies revealed that the SA‐bounded target system is more effective in suppressing the formation of iodine vacancy compared to other conditions (Figure 2G,H and Figure S16). These findings indicate that SA effectively contributes to the suppression of iodine vacancy on the perovskite surface.

Optical absorbance confirms a narrow bandgap of 1.25 eV (Figure S17), ideal for tandem photovoltaics. The Urbach energy (*E*_u_) [26], reflecting electronic disorder, decreased from 56 to 49 meV with SA, indicating lower defect density (Figure S18). To gain a deeper understanding of the effect of SA on Sn─Pb perovskite thin films, photoluminescence quantum yield (PLQY) analysis was conducted to investigate defects and non‐radiative recombination. The absolute external PLQY can be directly determined by calculating the ratio of the emitted photon flux to the absorbed photon flux using the following equation.

(1) $$\mathrm{PLQY}\;=\frac{\phi \mathrm{em}}{\phi \mathrm{abs}}=\frac{J_{rad}}{J_{G}}=\frac{J_{rad}}{J_{R}}=\frac{J_{rad}}{J_{\mathrm{rad}}+J_{\mathrm{non}}}\;$$

where *ϕ*_em_ is the emitted photon flux, *ϕ*_abs_ is the absorbed photon flux, 𝐽_G_ is the generation current, 𝐽_R_ is the total recombination current, 𝐽_𝑟𝑎𝑑_ is the radiative recombination current, and 𝐽_non_ is the nonradiative recombination current [27]. As shown in Figure 2I, SA‐treated films show an average PLQY increase from 4.07 × 10^−4^ (control) to 1.11 × 10^−3^ (Figure 2I and Table S1), reflecting reduced non‐radiative recombination and minimized *V*_OC_ losses. Steady‐state photoluminescence (SS‐PL) reveals enhanced intensity with SA, confirming fewer defects (Figure S19). Time‐resolved PL (TR‐PL) shows a longer carrier lifetime in SA‐treated films (600.8 vs. 495.68 ns for control; Table S2), indicating improved charge dynamics [28]. In layered structures (FTO/Al‐NiO_x_(ANO)/MA‐free Sn─Pb perovskite), SA‐treated films exhibit lower PL intensity and faster decay (14.76 vs. 20.48 ns for control) as shown in Figure S20 and Table S3, signifying superior charge extraction efficiency. These improvements, driven by SA's coordination (Section 2.1), improved crystallinity (Figure S9), and optimized morphology (Figure 2A–D) underscore its role as a bioavailable additive for high‐quality MA‐free Sn─Pb perovskite films. The improved optical properties of MA‐free Sn─Pb perovskite films can greatly enhance the device performance, particularly *V*_OC,_ due to minimized non‐radiative recombination.

### 2.3 Antioxidant Effect of Sinapic Acid

To evaluate the antioxidant properties of SA, we prepared MA‐free Sn─Pb perovskite precursor solutions with (target) and without (control) SA, stored under ambient conditions. Photographs taken over 24 h reveal distinct stability profiles (Figure S6). Initially, both solutions appeared bright yellow. Within 30 min, the control solution darkened, indicating Sn^2+^ oxidation to Sn^4+^ due to Sn's reactive 5s electrons compared to Pb^2+^. In contrast, the SA‐containing target solution retained its original color, demonstrating SA's robust antioxidant capability. SA's functional groups (COO─, ─OCH_3_, ─OH) donate electrons to neutralize oxidative species, stabilizing Sn^2+^ and Pb^2+^ in the solution. To further clarify the antioxidant contribution of the functional groups in SA, we compared SnI_2_ solutions containing SA, benzoic acid (BA), and tyrosol under identical air‐exposure conditions by UV–vis spectroscopy (Figure S21). The pristine SnI_2_ solution showed the progressive emergence of two oxidation‐related absorption bands at approximately 293 and 363 nm, whereas these bands were almost completely suppressed in the SA‐containing solution [29]. By contrast, BA‐ and tyrosol‐containing solutions showed only partial suppression of these features, indicating that each analogue alone provides limited protection. These new analogue‐controlled UV–vis experiments indicate that SA most effectively suppresses oxidation of SnI_2_ under air exposure among the tested molecules, whereas benzoic acid and tyrosol provide only partial protection. Consistent with this trend, the SnI_2_+SA solution retained its initial yellow color for the longest time during air exposure, while the pristine, BA‐containing, and tyrosol‐containing solutions turned deep red more rapidly (Figure S22). These results support a cooperative role of the carboxylate‐ and phenolic‐derived functionalities in SA and are in line with the known antioxidant behavior of phenolic acids.

X‐ray photoelectron spectroscopy (XPS) analysis of Sn 3d core spectra quantified Sn oxidation in control and target perovskite films (Figure 3A and Figure S23). The target film exhibited a significantly lower average Sn^4+^ ratio (23.66% in target vs. 54.28% in control; Table S4), confirming SA's suppression of Sn^2+^ oxidation. After 24 h of ambient exposure, the Sn^4+^ content in the target film remained consistently lower than in the control (Figure 3B). This enhanced stability aligns with SA's preferential coordination with Sn^2+^, as established in Section 2.1 (Figure 1C–E). To gain deeper insight into the spatial distribution and chemical interactions of functional moieties, we systematically performed XPS depth profiling on both control and additive sinapic acid (SA)‐treated perovskite films (Figures S24–S26, S28 and Tables S5 and S6). On the one hand, we also conducted time‐of‐flight secondary ion mass spectrometry (ToF‐SIMS) depth profiling on ITO/HTL/Sn─Pb perovskite films incorporating SA as shown in Figure S8. From the results, it was confirmed that SA is mainly distributed at the beneath perovskite film and HTL/perovskite interface. Following the ToF‐SIMS results, we fitted the depth profile patterns of C 1s, O 1s (Figures S25, S26, and Table S5), and Sn 3d (Figure S28 and Table S6) at 0, 100, 200, 300s. The region at 300s represents the beneath perovskite film and HTL/perovskite interface. And we clarify the distinct contributions of sinapic acid's two functional groups, the carboxylate (COO^−^) and the phenolic ‐OH. Both groups play complementary roles at different locations in the film, as evidenced by our XPS depth‐profile analysis. Together, they synergistically mitigate Sn^2+^ oxidation, passively develop defects, and help improve crystallization.

**FIGURE 3:**
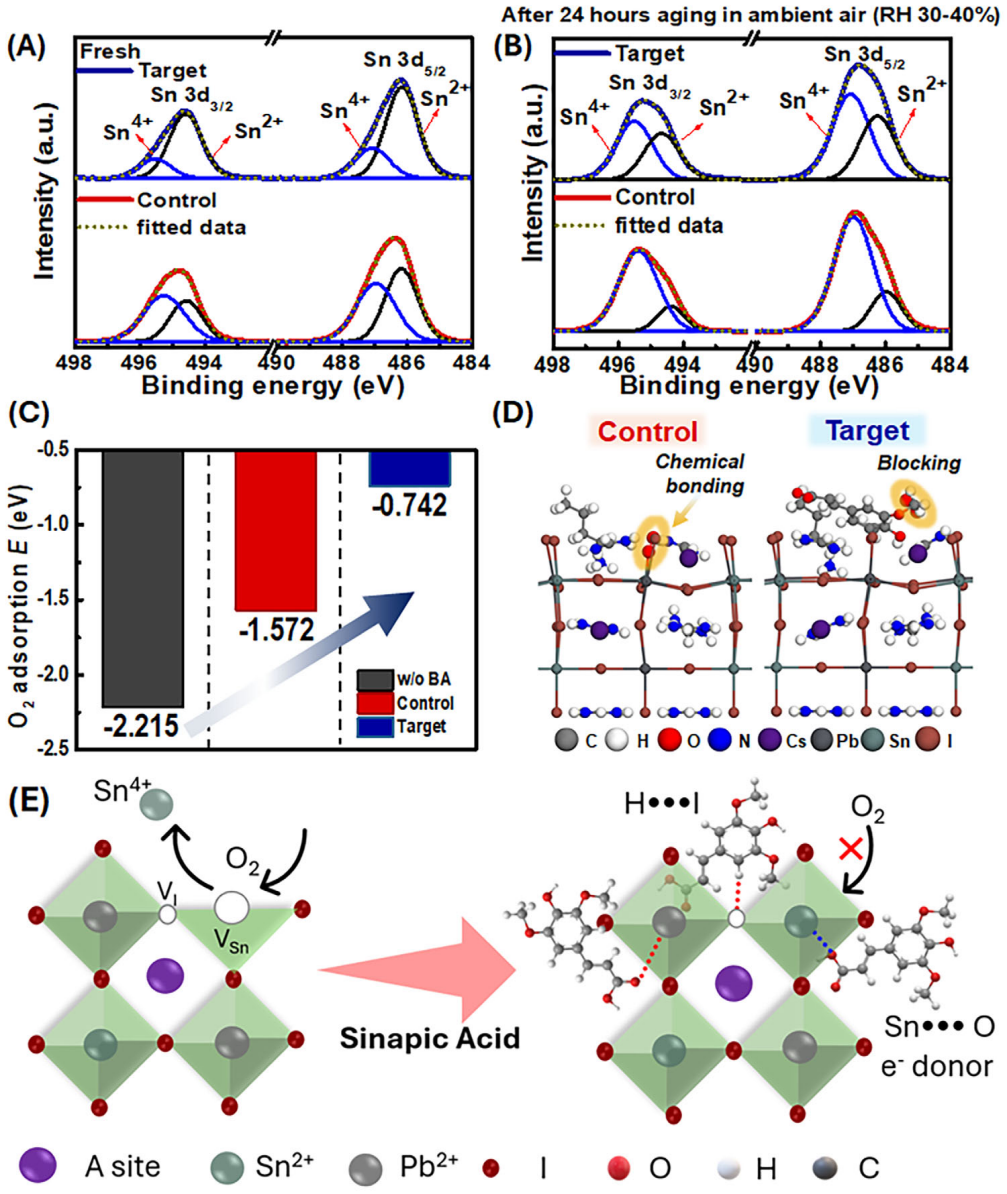
(A) Sn 3d XPS spectra of the fresh control and target perovskite films. (B) Sn 3d XPS spectra of the control and target perovskite films after 24 h aging in ambient air. (C) O_2_ adsorption energies of w/o BA, control, and target systems. (D) Optimized structures of control and target systems, illustrating the adsorption behavior of O_2_ molecule. (E) Schematic illustration of plausible defects in Sn─Pb halide perovskite, chemical interactions of SA with the perovskite that inhibit deep defects and suppresses Sn^2+^ oxidation.

Figure S25 shows the C 1s and O 1s spectra of control films at etching times from 0–300 s. At 0 s, the C 1s peak at 284.6 eV corresponds to adventitious carbon (calibration), while peaks at 287.8 eV and 288.9 eV arise from FA (C─N/C─NH_2_) and oxygenated carbon (C═O), respectively. At 100 s, a similar C─N/C─NH_2_ peak (287.9 eV) was detected; however, no such carbon signal was detected upon further etching from 200s to 300s [30, 31, 32, 33]. The O 1s spectra were deconvoluted into peaks at 529.4 eV (Pb─O/Sn─O), 530.7 eV (Sn─O_2_), and 532.8 eV (chemisorbed oxygen/hydroxyl) at the control perovskite surface. These signals disappeared after 100–200s etching but reappeared at 300s, indicating perovskite oxidation during etching [34, 35, 36]. In the target perovskite (Figure S26), the surface C 1s spectrum at 0 s etching deconvolutes into peaks at 284.6 eV (adventitious carbon), 285.7 and 287.6 eV (C─N/C─NH_2_ from FA^+^), and 289.4 eV (C═O from sinapic acid and oxygenated carbon generated during etching). An additional 291.4 eV peak, assigned to *π*–*π* interactions from the sinapic acid phenyl ring, provides direct evidence of its incorporation in the film [37]. In the target perovskite at 300 s etching, C 1s peaks at 284.6 eV (C─C/C═C), 286.0 eV (C─O─C), and 287.3 eV (C═O) were detected, corresponding to sinapic acid functionalities. Their presence near the interface suggests chemical interaction with the perovskite bulk and a role in promoting preferential growth orientation [32, 37, 38].

O 1s depth profiling of the target perovskite (Figure S26) revealed surface peaks at 529.5 eV (Pb─O/Sn─O, Sn^2+^), 530.5 eV (Sn─O_2_, Sn^4+^), 531.4 eV (C═O from sinapic acid), 532.5 eV (C─O─C/chemisorbed O), and 533.1 eV (─OH from sinapic acid). Signals disappeared between 100–200 s etching but reappeared at 300 s, with peaks at 529.8 eV (Pb─O/Sn─O) and 530.7 eV (Sn─O_2_). The Pb─O/Sn─O ratio was significantly higher in the target (32.62%) than in the control (13.0%), while Sn─O_2_ content was lower (36.73%) compared to the control perovskite (67.87%), as depicted in Table S5. Additional peaks at 531.7 and 533.4 eV correspond to C═O and ─OH/C─O functionalities of sinapic acid [39, 40].

Taken together, the depth‐profile XPS results indicate that integral SA molecules contribute cooperatively to oxidation suppression and defect passivation within the perovskite film. In the O 1s spectra of the SA‐treated target film, components at ∼531.4–531.7 eV and ∼533.1–533.4 eV are assigned to the C═O/COO^−^ and ─OH functionalities of SA, respectively, confirming the presence of SA‐related chemical species at both the film surface and the buried interface. The C═O group oxygen, which possesses a higher negative charge (−0.4934 e, Figure S27), can strongly coordinate with undercoordinated Sn/Pb centers, thereby stabilizing Sn^2+^ and suppressing its oxidation to Sn^4+^. Consistently, at 300 s sputtering, corresponding to the buried perovskite/HTL region, the SA‐treated target film exhibits a higher Sn^2+^ fraction (∼35.16%) and a lower Sn^4+^ fraction (∼21.45%) than the control film (Sn^2+^∼13.48%, Sn^4+^∼39.39%) (Figure S28 and Table S6). This confirms that SA effectively preserves Sn^2+^ and reduces Sn^4+^ formation near the buried interface. In addition to coordination by the C═O group, the phenolic ─OH group (−0.1066 *e* from Figure S27) of SA contributes to antioxidant protection through its electron‐/hydrogen‐donating ability. However, this role should be understood as part of the cooperative antioxidant function of the intact SA molecule, not as an independent function occurring in a spatially separated region. Therefore, SA suppresses Sn oxidation through a combined mechanism involving Sn/Pb coordination by oxygen‐containing groups and antioxidant protection from the phenolic moiety, leading to reduced interfacial oxidation and improved chemical stability of the perovskite film. Together, they synergistically mitigate Sn^2+^ oxidation, passivate defects, and help improve crystallization.

To elucidate the mechanism in terms of the computational aspect, we performed density functional theory (DFT) calculations to assess O_2_ adsorption on perovskite surfaces with iodine vacancies (Figure 3C,D and Figure S29). In the control system, O_2_ binds strongly to vacancy sites, promoting oxidation. Conversely, SA‐treated surfaces exhibit thermodynamically unfavorable O_2_ adsorption, as SA passivates defect sites, hindering oxidative interactions. This corroborates the reduced Sn^4+^ content observed in XPS, highlighting the role of SA in contributing to effective antioxidant behavior to prevent oxygen‐induced degradation. We propose a defect passivation mechanism for Sn─Pb perovskites, illustrated in Figure 3E. In control films (Figure 3E, left), uncoordinated Sn^2+^ cations, arising from ion migration, oxidize to Sn^4+^, forming deep trap states that drive non‐radiative recombination and accelerate degradation. In contrast, SA‐treated films (Figure 3E, right) leverage SA's multifunctional groups to form stable interactions with undercoordinated Sn^2+^/Pb^2+^ ions (e.g., COO^−^···Sn/Pb, CH_3_O···Sn/Pb). The ─OH group's electron‐donating capacity further inhibits oxidation, through hydrogen bonding (H···I) with halide ions and suppresses iodine vacancy formation. These interactions create a Lewis acid‐base adduct network that stabilizes precursors, regulates crystallization (Section 2.1), and enhances film morphology (Section 2.2).

SA's incorporation into the final perovskite film, confirmed by prior analyses, reduces defect density and oxidative susceptibility. This multifaceted passivation combining chemical coordination, vacancy suppression, and antioxidant protection enhances device stability and photovoltaic performance. By leveraging a bioavailable additive, our approach not only mitigates Sn─Pb perovskite's intrinsic instability but also contributes to enhanced device stability and overall photovoltaic performance.

### 2.4 Photovoltaic Device Characterization for MA‐Free Sn─Pb PSCs with Sinapic Acid

Figure 4A depicts the schematic illustration of the MA‐free Sn─Pb PSCs with a p‐i‐n layered structure of FTO/ANO nanocrystals (HTL)/perovskite/PC_61_BM (ETL)/BCP (buffer layer)/Ag as described in the experimental section of supplementary information. To examine a change in the band alignment of the perovskites upon addition of SA, Ultraviolet photoemission spectroscopy (UPS) measurements were conducted (Figure S30). The binding energy (*E*_B_) values at the cutoff and valence band (VB) region are estimated to be 0.44 and 16.42 eV for the control perovskite and 0.63 and 16.41 eV for the target perovskite, respectively. Based on these UPS results, the corresponding energy level diagrams for the control and target devices were depicted (Figure S31). Upon the introduction of SA, the VB level shifted from −5.22 to −5.42 eV, while the conduction band level moved from −3.97 to −4.17 eV. This implies a well‐aligned energy band, which facilitates more efficient charge extraction to the ANO NC HTL and PC_61_BM ETL [22], ultimately resulting in a higher *V*_OC_. The current density–voltage (*J–V*) curves for both the control and target devices are shown in Figure 4B and the corresponding photovoltaic parameters are summarized in Table 1. With the incorporation of the SA molecule, the efficiency of the PSCs improved from 20.7% to 23.2%, positioning this work among the highest PCE reported for MA‐free Sn─Pb PSCs (Table S7). Notably, the *V*_OC_ increased from 0.827 to 0.893 V, marking one of the highest *V*_OC_ values reported for MA‐free Sn─Pb PSCs to date. This enhancement is conjectured to be related to the suppression of defect density and non‐radiative recombination. To further benchmark the effectiveness of SA against structurally related phenolic additives, we performed side‐by‐side device comparisons using gallic acid (GA) and caffeic acid (CA) under the same device architecture. The GA‐treated device exhibited a PCE of 21.26% with a *V*_OC_ of 0.861 V, a *J*_SC_ of 32.88 mA/cm^2^, and an FF of 75.19%, whereas the CA‐treated device yielded a PCE of 20.01% with a *V*_OC_ of 0.840 V, a *J*_SC_ of 33.23 mA/cm^2^, and an FF of 71.70% (Figure S32). In comparison, SA delivered the best overall performance, with particularly clear advantages in *V*_OC_, FF, and PCE. This difference is likely associated with the distinct molecular structure of SA, which combines a hydroxycinnamic‐acid backbone with a carboxyl group, one phenolic hydroxyl group, and two methoxy groups, thereby providing a more balanced combination of precursor coordination, oxidation suppression, and defect passivation. By contrast, GA lacks the hydroxycinnamic backbone and methoxy functionalities, while CA, although sharing the hydroxycinnamic backbone, does not possess the same methoxy‐substituted motif as SA. These structural differences are consistent with our mechanistic results showing that SA effectively interacts with Sn/Pb precursors, suppresses Sn^2+^ oxidation, and reduces non‐radiative recombination, so that its benefit in this MA‐free Sn─Pb/NiO_x_ platform is expressed primarily through reduced voltage loss and improved fill factor rather than through a major change in *J*_SC_. Figure S33 exhibits the external quantum efficiency (EQE) spectra for both control and target devices. The integrated short circuit current density (*J*_SC_) values calculated from EQE were 32.78 and 32.93 mA/cm^2^ for the control and target devices, respectively, which are in good agreement with those derived from *J–V* measurements. This confirms that the introduction of the SA molecule has a greater impact on improving *V*_OC_ than *J*_SC_. Steady‐state photocurrent measurements were conducted for 600 s at the maximum power point voltage (*V*_mpp_) of 0.72 V (control) and 0.77 V (target), as depicted in Figure S34. While the control device showed a slight decrease in photocurrent, the target device exhibited stable photocurrent retention. Furthermore, the target device displayed minimal hysteresis compared with the control device (Figure S35), which is consistent with the reduced trap density, suppressed non‐radiative recombination, and improved compact morphology induced by SA. In addition, the target device achieved a high fill factor (FF) of 81% (Figure S36), ranking among the highest reported MA‐free Sn─Pb PSCs, particularly those utilizing NiO_x_‐based HTL. Device reproducibility was also assessed across 20 fabricated devices. The statistical distribution of photovoltaic parameters for the control and target devices is presented in Figure 4C, Figure S37, and Tables S8 and S9. Interestingly, the target device with SA showed an impressive reproducible performance with average values of 0.885 ± 0.007 V for *V*_OC_, 77.3% ± 1.51% for FF, and 22.7% ± 0.22% for PCE. To evaluate whether the beneficial effect of SA is retained upon area scaling, we further fabricated and tested 1 cm^2^ devices under the control and target conditions (Figure S38 and Table S10). The 1 cm^2^ control device exhibited a *V*_OC_ of 0.817 V, *J*_SC_ of 30.80 mA/cm^2^, FF of 64.52%, and PCE of 16.23%, whereas the 1 cm^2^ target device delivered a *V*_OC_ of 0.890 V, *J*_SC_ of 31.19 mA/cm^2^, FF of 70.35%, and PCE of 19.53%. Statistics from five 1 cm^2^ devices further confirmed the reproducibility of this larger‐area performance (Table S11), with average *V*_OC_ and PCE values of 0.813 ± 0.004 V and 15.74% ± 0.30% for the control and 0.886 ± 0.003 V and 19.43% ± 0.07% for the target, respectively. Importantly, the SA‐treated target maintained a high *V*_OC_ of 0.890 V even at the larger active area and showed clear improvements in FF and PCE over the large‐area control, indicating that the beneficial effect of SA is preserved beyond the small‐area. A significant improvement in device performance is primarily attributable to an enhancement in both *V*_OC_ and FF upon the incorporation of SA. Hence, we first focus on the origin of improved *V*_OC_. To elucidate how SA incorporation influences the charge recombination mechanism, we measured the light intensity dependence of *V*_OC_ (Figure 4D). Based on the equivalent circuit model under typical condition, *V*_OC_ is given by following equation:

(2) $$qV_{\mathrm{oc}}=n_{id}\;kTln\left(\frac{J_{\mathrm{SC}}}{J_{0}}\right)$$

where *n*_id_ is the ideality factor, *k*_B_ is the Boltzmann constant, *q* is the elementary charge, *T* is the temperature, and *J*_0_ is the reverse saturation current density [12, 41]. From the slope in plots of *V*_OC_ against the logarithm of *J*_SC_, *n*_id_ was estimated to be approximately 1.4 for the control device and 1.2 for the target device. This suggests that the incorporation of SA in MA‐free Sn─Pb perovskites effectively reduces trap‐assisted recombination, eventually leading to higher *V*_OC_ and FF. Figure 4E shows the electrochemical impedance spectroscopy (EIS) results measured in the dark over a frequency range from 1 Hz to 1 kHz. The recombination resistance (*R*_rec_), which is directly associated with the parallel resistance, was remarkably higher in the target device than in the control device, demonstrating suppressed non‐radiative recombination.

**FIGURE 4:**
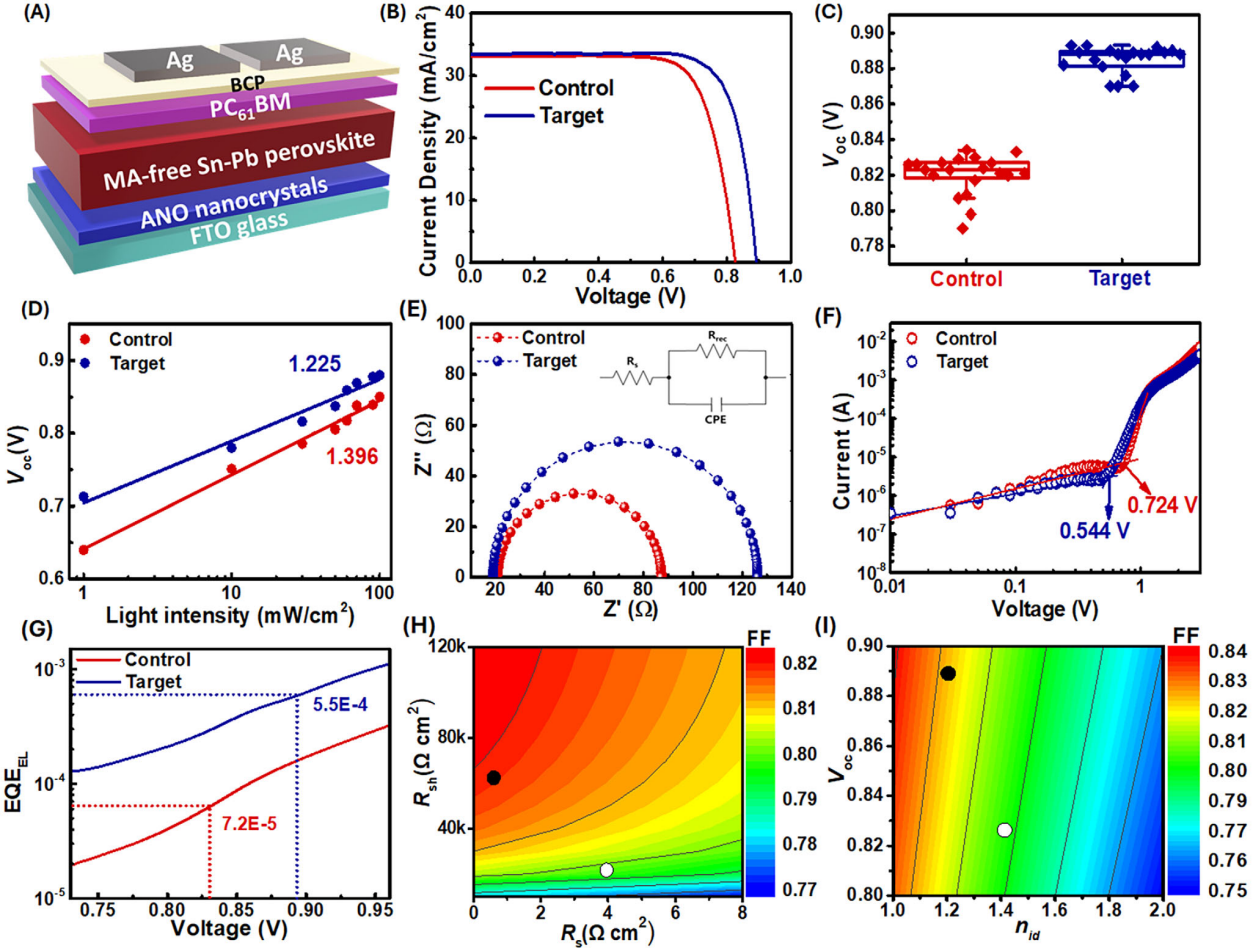
(A) Schematic illustration of the MA‐free Sn─Pb perovskite device structure. (B) *J–V* curves for control and target devices. (C) Box charts illustrating the photovoltaic parameters of *V*_OC_ for 20 devices. (D) Light intensity‐dependent *V*_OC_ for the devices. (E) Nyquist plots of the PSCs for control and target conditions. (F) Dark *I–V* characteristics of a hole‐only device with FTO/ANO/perovskite/P3HT/Ag structure. (G) EQE_EL_ as a function of the injection voltage to calculate the nonradiative recombination loss. (H) Contour map of FF calculated by Equation (5) using different *R*_s_ and *R*_sh_ resistances with *n*_id_ and *V*_OC_ values from the control devices as fixed parameters. White circle and black circle represent the control device and target device, respectively. (I) Contour map of FF calculated by Equation (5) using different *n*_id_ and *V*_OC_ with *R*_s_ and *R*_sh_ values from the control devices as fixed parameters. White circle and black circle represent the control device and target device, respectively.

**TABLE 1:** Photovoltaic parameters of the PSCs for control and target under AM 1.5G illumination.

	*V*_OC_ (V)	*J*_SC_ (mA/cm^2^)	FF (%)	PCE (%)
Control	0.827	33.23	75.2	20.7
Target	0.893	33.42	77.7	23.2

Next, to quantify the trap density, space charge limited current (SCLC) measurements were conducted using hole‐only devices (FTO/ANO/perovskite/P3HT/Ag) as shown in Figure 4F [42]. The trap density values were estimated to be 5.6 × 10^15^ cm^−3^ for the control device and 4.2 × 10^15^ cm^−3^ for the target device. The lower trap density in the target device indicates a reduction in defect sites, which primarily contributes to an increase in *V*_OC_ and FF.

To further evaluate the voltage loss caused by nonradiative recombination, we measured the EQE of electroluminescence (EQE_EL_) from the devices. Figure 4G shows EQE_EL_ as a function of applied voltage for both the control and target devices. The target device displayed a significantly enhanced EQE_EL_ of about 5.5 × 10^−4^ at 0.89 V, which is roughly eight times higher than that of the control device (∼7.2 × 10^−5^ at 0.83 V). The nonradiative (∆*V*_nr_) was calculated from the measured EQE_EL_ values using the following equation [27, 43]:

(3) $$\Delta V_{\mathrm{nr}}=\frac{kT}{q}\;n\left({\mathrm{EQE}}_{\mathrm{EL}}^{-1}\right)$$

The ∆*V*_nr_ values were estimated to be approximately 0.247 V for the control device and 0.194 V for the target device, indicating that the voltage losses due to nonradiative recombination were notably suppressed in the target device. Interestingly, the difference in ∆*V*_nr_ between the two devices directly correlates with variations in *V*_OC_ and hence the overall PCE. Hence, this improvement can be ascribed to enhanced defect passivation and more efficient charge extraction at the interfaces upon the incorporation of the bioavailable phytophenol SA molecule. Furthermore, in Figure S39, we performed the quantitative analysis for voltage loss based on the detailed balance theory, known as the Rau analysis [44]. The detailed process for evaluation is described in the supporting information. As summarized in Table S12, a notable difference of 0.06 was observed in ∆*V*_nr_, while ∆*V*_SC_ and ∆*V*_rad_ were negligibly small. These results align well with the ∆*V*_nr_ values derived from EQE_EL_ measurement. This result strongly suggests that the improvement in *V*_OC_ and overall device performance is primarily ascribed to the suppression of nonradiative recombination through the incorporation of the SA molecule into Sn─Pb perovskite.

The beneficial impact of SA incorporation was also reflected in the increased FF. Therefore, we further investigated the underlying cause of this enhancement by analyzing FF based on an empirical equation [45, 46, 47]. In an ideal photovoltaic device in which series resistance (*R*_s_) is nearly zero (*R*_s_ ≈ 0) and shunt resistance (*R*_sh_) approaches infinity (*R*_sh_ → ∞), the FF can be expressed as [48]

(4) $$F\;F_{0}=\frac{v_{\mathrm{OC}}-\ln\left(v_{\mathrm{OC}}+0.72\right)}{v_{\mathrm{OC}}+1}\;$$

where *v*_OC_ represents the dimensionless voltage, defined as *v*_OC_ = *qV*_OC_/*n*_id_ *k*_B_ *T*. However, in real solar cells, the FF is influenced by both *R*_s_ and *R*_sh_ and hence is typically expressed as

(5) $$\mathrm{FF}\;=\;FF_{s}\left(1-\frac{v_{\mathrm{OC}}+0.7}{v_{\mathrm{OC}}}\frac{FF_{s}}{r_{\mathrm{sh}}}\right)$$

where FF_s_ is given by

(6) $$F\;F_{s}=FF_{0}\left(1-1.1r_{s}\right)+\frac{{r_{s}}^{2}}{5.4}$$

Here, *r*_s_ and *r*_sh_ denote the normalized resistances, defined as *r*_s_ = *J*_SC_ *R*_s_/*V*_OC_ and *r*_sh_ = *J*_SC_ *R*_sh_/*V*_OC_, respectively. Using Equations ((4), (5), (6)), the FF values for PSCs with and without the SA molecule were evaluated based on experimental data. As summarized in Table S13 the calculated FF values closely match the experimentally obtained FF values, with an error margin of less than ∼8%. This allows us to systematically analyze how each parameter contributes to variations in FF. While the difference in ​*J*_SC_ was minimal between the two devices, significant variations were found in *n*_id_, *V*_OC_, *R*_s_, and *R*_sh_. We hence plotted the contour map of FF as a function of either *n*_id_ and *V*_OC_ or *R*_s_ and *R*_sh_ in Figure 4H,I. These maps were constructed using Equations ((4), (5), (6)) with *R*_s_ and *R*_sh_ or *n*_id_ and *V*_OC_ values from the control devices as fixed parameters, respectively. As shown in Figure 4H,I, FF is highly sensitive to variations in *n*_id_, *V*_OC_, *R*_s_ and *R*_sh_. The relatively contributions of these parameters to FF enhancement are summarized in Table S14, revealing the following order of influence: *n*_id_ > *R*_sh_ > *V*_OC_ > *R*_s_. In summary, the enhancement in FF is mainly ascribed to a reduction in defects (as reflected in *n*_id_ and *V*_OC_) and partly due to changes in resistance components (as reflected in *R*_s_ and *R*_sh_). This strongly suggests that the incorporation of SA molecules not only effectively passivates defects within the perovskite bulk but also promotes a more uniform and void‐free perovskite morphology, resulting in increased FF and hence device performance.

### 2.5 The Device Stability of MA‐Free Sn─Pb Perovskite Solar Cells with Sinapic Acid

To investigate the impact of SA on the stability of MA‐free Sn─Pb perovskite films, we evaluated phase stability using XRD over time under ambient conditions (35% relative humidity, RH). XRD patterns after one day revealed that the control film (without SA) exhibited a significant reduction in peak intensities for the (100) and (200) perovskite planes compared to the SA‐treated target film (Figure 5A,B). This indicates that SA effectively mitigates Sn^2+^ oxidation and defect formation, enhancing phase stability. TGA further elucidated SA's role in thermal stability (Figure S7). SA‐incorporated perovskite powders displayed a higher sublimation temperature than pristine perovskite powders, suggesting stronger interactions between SA and perovskite precursors. This increased activation energy for thermal decomposition implies that SA enhances the thermal endurance of the perovskite structure.

**FIGURE 5:**
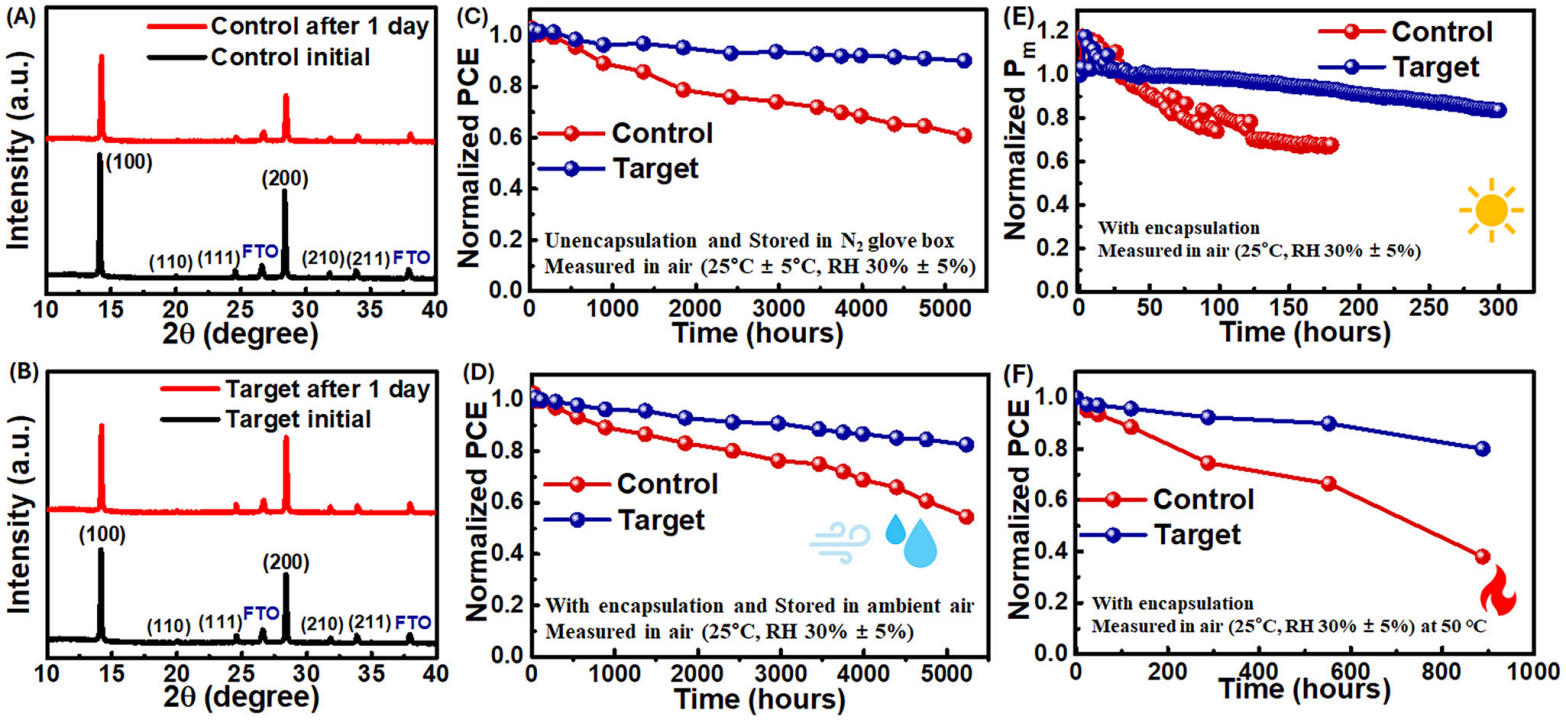
Time‐dependent XRD patterns of (A) the control and (B) target perovskite films. (C) Long‐term stability of the MA‐free Sn─Pb PSCs for control and target without encapsulation stored in a nitrogen atmosphere. (D) Long‐term stability of the encapsulated PSCs corresponding conditions in ambient air. (E) The operational stability of the encapsulated devices under maximum power point tracking (MPPT) and 1 sun AM1.5G illumination in ambient air. (F) Thermal stability of the encapsulated devices.

Unencapsulated devices were stored continuously in an N_2_‐filled glovebox between measurements, while encapsulated devices were stored in ambient air. In both cases, long‐term stability was tracked by periodically measuring device performance under ambient laboratory conditions (25°C ± 5°C, RH 30% ± 5%) over 5200 h (Figure 5C,D). The target device with SA achieved unprecedented stability, retaining over 91% of its initial power conversion efficiency (PCE), comparable to the recently reported stability for MA‐free Sn─Pb PSCs (Table S7). In contrast, the control device degraded to 65% of its initial PCE. For encapsulated devices in air, the target device maintained 85% of its initial PCE, while the control retained only 61%. Operational stability under AM1.5G illumination at room temperature with maximum power point tracking (MPPT) showed the target device preserving over 84% of its initial PCE after 300 h, far outperforming the control (Figure 5E). The thermal stability of encapsulated devices was assessed at 50°C in the air for 900 h (Figure 5F). The target device retained 80% of its initial PCE, compared to 38% for the control. This exceptional stability is attributed to SA's suppression of Sn^2+^ oxidation, reduced defect density, minimized non‐radiative recombination, and enhanced phase stability, as corroborated by TGA and XRD analyses. To further assess the practical stability of larger‐area devices, we evaluated the long‐term stability of 1 cm^2^ control and target PSCs under unencapsulated air‐storage conditions (25°C ± 5°C, RH 30% ± 5%; Figure S40). The 1 cm^2^ target device exhibited markedly improved environmental stability compared with the large‐area control, retaining approximately 70% of its initial normalized PCE after 1000 h, whereas the control degraded much more rapidly to approximately 37% over the same period. This result indicates that the stabilizing effect of SA is maintained not only in small‐area devices but also under larger‐area conditions, which is fully consistent with the antioxidant, defect‐suppression, and phase‐stabilization characteristics already identified in the present work. To probe the origin of the improved large‐area stability, we further measured the XRD patterns of 1 cm^2^ devices before and after 30 days of air storage (Figure S41). The aged control device exhibited additional diffraction peaks assignable to AgI, and the inset photograph showed severe discoloration/degradation of the Ag electrode [49, 50]. In contrast, the aged target device did not show discernible AgI formation and retained a much less altered electrode appearance. Because AgI formation is associated with iodide migration toward the Ag electrode followed by interfacial reaction, these results indicate that the control undergoes much more severe halide migration and electrode‐side degradation than the SA‐treated target. This behavior is particularly consistent with our earlier defect‐passivation analysis, in which the SA‐bounded target system was found to suppress iodine‐vacancy formation more effectively than the control (Figure 2G,H and Figure S16). Since iodine‐vacancy defects are widely recognized as major migration pathways for halide ions in perovskite absorbers, the reduced AgI formation in the target device suggests that SA suppresses vacancy‐assisted iodide migration in addition to improving oxidative stability. Therefore, the absence of AgI formation in the target device provides additional macroscopic evidence that SA mitigates migration‐enabled degradation pathways and contributes to improved long‐term device stability. These results highlight the pivotal role of SA, a bioavailable phytophenol, in advancing the durability and performance of MA‐free Sn─Pb PSCs, paving the way for scalable, eco‐friendly photovoltaic technologies.

## 3 Conclusion

In this study, we developed highly efficient and stable MA‐free Sn─Pb PSCs by incorporating sinapic acid (SA), a bio‐derived phytophenol. Our findings elucidate the fundamental coordination mechanisms between SA and Sn─Pb perovskite precursors. SA plays a pivotal role in suppressing Sn^2+^ oxidation, enhancing crystallographic order, improving film morphology, and reducing defect density, as evidenced by comprehensive analyses. In addition, *V*oc loss is effectively suppressed by SA introduction, identified through a non‐radiative recombination study. Consequently, the champion MA‐free Sn─Pb PSC achieves a high PCE of 23.2% and a V_OC_ of 0.893 V among the highest reported for Sn─Pb PSCs. The device also demonstrates unprecedented long‐term stability, retaining over 91% of its initial PCE after 5200 h in both N_2_ and air environments. Furthermore, it exhibits remarkable operational stability, maintaining 84% of its initial performance after 300 h under continuous AM1.5G illumination. These advancements not only elevate the performance and durability of Sn─Pb PSCs but also unlock significant potential for their integration into tandem solar cells and narrow‐bandgap devices for near‐infrared applications, advancing eco‐friendly and scalable photovoltaic technologies.

## Author Contributions

S.L., P.P., and D.G.L. contributed equally to this work. S.L. and P.P. conceived the original concept and designed the experiments. D.G.L. conducted the DFT calculation for the research. S.L., P.P., and D.G.L. wrote the manuscript. T.‐G.L., S.Y. and I.N. carried out the characterization of films and devices. S.W.P. supported the DFT calculation. J.R., S.W.C., and D.‐G.L. prepared materials and conducted corresponding characterizations. H.D.K., T.K.L., and D.‐W.K. supervised the research, provided financial support, and revised and polished the manuscript. All authors discussed the results and reviewed the manuscript.

## Conflicts of Interest

The authors declare no conflicts of interest.

## Supporting information




**Supporting File 1**: advs76007‐sup‐0001‐SuppMat.docx.

**Supporting File 2**: advs76007‐sup‐0002‐Data.zip.

## Data Availability

The data that support the findings of this study are available from the corresponding author upon reasonable request.
