# Supplementary material for: Efficient and Stable MA‐Free Sn─Pb Perovskite Solar Cells With Exceptional V OC by Bioavailable Phytophenol Sinapic Acid
Source: Adv Sci (Weinh). 2026 Sep 1;13(53):e76007. doi: 10.1002/advs.76007 (PMC13534338; doi:10.1002/advs.76007)
Supplement: Supplementary file 1 [file ADVS-13-e76007-s002.docx]

Supporting Information

**Efficient and Stable MA-free Sn-Pb Perovskite Solar Cells with Exceptional *V*_OC_ by Bioavailable Phytophenol Sinapic Acid**

Seojun Lee^1, †^, Padmini Pandey^1, †^, Dong Gyu Lee^2, 3, †^, Tae-Gyeong Lee^1^, Saemon Yoon^1^, Insub Noh^4^, Sang Wook Park^5^, Jun Ryu^6^, SungWon Cho^6^, Dong-Gun Lee^6^, Hyung Do Kim^4,7 *^, Tae Kyung Lee^2, 3, *^ and Dong-Won Kang^1, *^

^1^ Department of Energy Systems Engineering, Chung-Ang University, 84 Heukseok-ro, Dongjak-gu, Seoul 06974, Republic of Korea.

^2^ Department of Organic and Nano Engineering, Hanyang University, 222 Wangsimni-ro, Seongdong-gu, Seoul 04763, Republic of Korea

^3^ Human-Tech Convergence Program, Hanyang University, 222 Wangsimni-ro, Seongdong-gu, Seoul 04763, Republic of Korea

^4^ Department of Polymer Chemistry, Graduate School of Engineering, Kyoto University, Katsura, Nishikyo-ku, Kyoto, 615-8510, Japan.

^5^ Department of Materials Engineering and Convergence Technology, Gyeongsang National University (GNU), 501 Jinju-daero, Jinju 52828, Republic of Korea

^6^ Department of Smart Cities, Chung-Ang University, 84 Heukseok-ro, Dongjak-gu, Seoul 06974, Republic of Korea.

^7^ Department of Materials Science and Engineering, Ajou University, Suwon, Republic of Korea

† Contributed Equally

* Corresponding Author: Dong-Won Kang ([kangdwn@cau.ac.kr](mailto:kangdwn@cau.ac.kr)), Tae Kyung Lee ([tklee@hanyang.ac.kr](mailto:tklee@hanyang.ac.kr)), Hyung Do Kim ([hyungdokim@photo.polym.kyoto-u.ac.jp](mailto:hyungdoKim@photo.polym.kyoto-u.ac.jp))

**Experimental section**

**Materials**

Formamidinium iodide (FAI, Greatcell Solar), *n*-butylammonium iodide (*n*-BAI, Greatcell Solar), Cesium iodide (CsI, 99.999 %, Alfa Aesar), lead(Ⅱ) iodide (PbI_2_, 99.99 %, Tokyo

Chemical Industry Co., Ltd.), tin(Ⅱ) fluoride (SnF_2_, 99 %, Sigma-Aldrich), Sn powder (99.8%, Sigma-Aldrich), iodine (I_2_, 99.8%, Sigma-Aldrich), and germanium iodide (GeI_2_, 99.8%, Sigma-Aldrich), sinapic acid (≥ 98%, Sigma-Aldrich) were purchased and used as precursors for Sn-Pb binary perovskites without purification. Ethane-1,2-diammonium iodide (EDAI_2_, Greatcell Solar) was purchased and used as passivation of Sn-Pb perovskite film surface. Dimethylformamide (DMF, 99.5 %, Samchun Chemical) and dimethyl sulfoxide (DMSO, 99.8 %, Samchun Chemical), toluene (99.8 %, Samchun Chemical), 2-Propanol (IPA, Sigma-Aldrich), and chlorobenzene (CBZ, 99 % - GR grade, Wako) were used as solvents. Acetone ( ≥ 99 %, Samchun Chemical) and Isopropanol (IPA, ≥ 99.5 %, Samchun Chemical) were used as cleaning solvents for Fluorine-doped tin oxide (FTO)-coated glasses. Poly(3,4-ethylenedioxythiophene) polystyrenesulfonate (PEDOT:PSS, PVP AI 4083) was sourced from CleviosTM (Germany). Phenyl-C_61_-butyric acid methyl ester (PC_61_BM, 99.5 %) was sourced from Organic Semiconductor Materials (OSM, Republic of Korea) and Bathocuproin (BCP, 98 %) was purchased from Alfa Aesar. Poly(3-hexylthiophene-2,5-diyl) (P3HT, 4002-EE, Mw = ca. 60 K, RR = ~ 90 %) was purchased from Rieke Metals. Nickel nitrate hexahydrate (Ni(NO_3_)_2_·6H_2_O), aluminum nitrate nonahydrate (Al(NO_3_)_3_·9H_2_O) were purchased from Sigma-Aldrich.

**Preparation of perovskite precursor solution**

1. Preparation of SnI_2_ solution

A SnI_2_ solution was prepared using Sn powder and I_2_. Initially, 0.8M I_2_ was dissolved in 0.2 mL of DMSO and stirred for 1 hour. Following this, 0.8 mL of DMF was added to the I_2_ solution dissolved in DMSO, and the mixture was stirred for an additional 10 min. Subsequently, Sn powder at a concentration of 0.8 M was introduced into the I­_2_ solution and the reaction mixture was stirred for overnight to ensure complete dissolution. The resulting SnI_2_ solution was then used for the preparation of a 1.6 M MA-free Sn-Pb perovskite in the desired stoichiometric ratio.

2. Preparation of 1.6 M MA-free Sn-Pb perovskite solution

A BA_0.02_(FA_0.83_Cs_0.17_)_0.98_Sn_0.5_Pb_0.5_I_3_ precursor solution with a concentration of 1.6 M was prepared using n-BAI, FAI, CsI, and PbI₂, maintaining a stoichiometric ratio of 0.83:0.17:0.5:0.5. Additionally, 0.08 M of excess SnF_2_, 0.056 M of Sn powder, and SnI_2_ solution were incorporated into the mixture. After preparing the components including *n*-BAI, FAI, CsI, PbI_2_, SnF_2_, Sn powder, and 1.042 mL of the filtered SnI_2_ solution was introduced into the prepared mixture. Sinapic acid (SA) was added 1 mol% in the 1.6M MA-free Sn-Pb perovskite precursor solution. The MA-free Sn-Pb perovskite precursor solution was obtained after stirring for 2 hours.

**Device Fabrication**

FTO-coated glass substrates (Asahi) were sequentially cleaned in an ultrasonic bath using acetone and isopropanol (IPA) for 20 min each. Following the cleaning process, the substrates were dried in a 95 °C oven to remove any residual solvents. Subsequently, the dried substrates were then subjected to UV treatment for 30 min. Al-NiO_x_ (ANO) nanocrystal synthesis method for hole transport layer (HTL) was presented in our previous work [1]. A 50 μL of ANO NC was spin-coated onto FTO-coated substrates under ambient conditions using a two-step process: 1000 rpm for 1 s followed by 3000 rpm for 30 s. The coated substrates were then annealed at 150 °C for 20 minutes on a hotplate to form an inorganic hole transport layer (HTL) with a thickness of approximately 10–20 nm. After annealing, the HTL-coated substrates were transferred into N_2_-filled glove box. The perovskite precursor solutions with or without sinapic acid were spin-coated at 5000 rpm for total of 35 s in the N_2_-filled glove box. During the spin-coating process, 300 μL of toluene was dropwise applied at the center of the substrate between 12 and 13 s. The perovskite-coated substrates were then annealed sequentially at 65 °C for 1 min and 100 °C for 10 min. For surface passivation, an EDAI_2_ solution (0.5 mg/mL in IPA) was dynamically spin-coated at 5000 rpm for 30 s, followed by annealing at 100 °C for 5 min to remove residual solvents. The electron transport layer (ETL) was formed by spin-coating a PC_61_BM solution (20 mg/mL in CBZ) onto the perovskite film at 1500 rpm for 35 s, followed by annealing at 75 °C for 10 min. A BCP solution (0.5 mg/mL in IPA) was then spin-coated at 4000 rpm for 20 s without any additional annealing. The silver (Ag) top electrodes (ca. 180 nm) were thermally evaporated under a high vacuum condition (< 2 × 10^-6^ Torr) using a shadow mask, with each solar cell device having an active area of 4 mm^2^.

The hole-only devices for space-charge limited current (SCLC) were fabricated with a structure of FTO/ANO/perovskite/P3HT/Ag. The fabrication process followed the same steps as perovskite solar cells (PSCs), except for the inclusion of a P3HT layer. A P3HT solution (10 mg/mL in CBZ) was spin-coated onto the perovskite film at 3000 rpm for 30 s, followed by annealing at 100 °C for 5 min.

**Film characterization**

The optical absorption spectra were characterized using ultraviolet-visible (UV–Vis) spectroscopy (UV-2700, Shimadzu). The steady-state photoluminescence (SS-PL) and time-resolved PL spectra were measured using a spectrofluorometer (Fluorolog3 with TCSPC, HORIBA SCIENTIFIC) with a laser excitation wavelength at 463 nm. The crystal structure of the perovskite films was evaluated by X-ray diffraction (XRD, D8-Advance, Bruker-AXS). A diffracted beam monochromator was equipped in the X-ray diffractometer (Cu Kα radiation, λ = 1.541 Å). All the XRD data were recorded in the two-theta range from 10° to 40° with a 1° min^−1^ scanning rate at RT. The surface morphologies and cross-sectional images were investigated by field emission scanning electron microscopy (FE-SEM; AURIGA, Carl Zeiss). X-ray photoelectron spectroscopy (XPS) measurement was performed with a K-alpha system (Thermo Fisher Scientific) using a monochromatic Al Kα irradiation (1486.6 eV). Ultraviolet photoelectron spectroscopy (UPS) measurement was performed using an XPS-Theta Probe (Thermo Fisher Scientific) with Al Kα radiation (1486.6 eV). For the measurement of relative photoluminescence quantum yield (PLQY), photoluminescence spectra were obtained under continuous wave excitation using a 532 nm diode laser. A BEL-300 spectrometer (Bunkoukeiki) was used to record the spectra. All measurements were carried out in ambient air with glass-encapsulated samples. The integrated PL intensity was recorded at varying excitation intensities, and the relative PLQY was defined as PLQY=I_PL_/I_pump_. Absolute PLQY was obtained from measurements performed in an integrating sphere with thin film samples deposited on glass and encapsulated in glass. PLQY values were calculated employing the method proposed by de Mello et al using the equations: $PLQY=\frac{P_{c}-(1-A)P_{B}}{L_{A}A}$ and $A=1-\frac{L_{C}}{L_{B}}$, P_B_ and P_C_ represent the integrated intensities of diffused PL when the sample is inside the sphere but outside the laser beam path and when it is directly illuminated by the laser, respectively. L_A_, L_B_ and L_C_ are the integrated intensities of the excitation light when the sample is outside the sphere, inside the sphere but outside the laser beam path, and inside the sphere under direct laser illumination, respectively [2]. This method provides reliable PLQY determination for thin film samples under controlled experimental conditions.

**Device testing**

All device characterizations were carried out in ambient conditions (RT and relative humidity (RH) of 25 – 30 %). Current-voltage (*J–V*) curves of the perovskite solar cells were measured using a solar simulator (PEC-L01, Peccell Technologies) under standard AM 1.5 illumination (power, 100 mW/cm^2^). The maximum power point tracking (MPPT) was measured under 100 mW/cm^2^ illumination using LED solar simulator (Newport, LSH-7320). The light intensity was calibrated with a silicon photodiode detector (BS-500BK, BUNKOUKEIKI CO., LTD.). Each device was measured with a scan rate of 100 mV/s^−1^ using a Keithley 2400 source meter. The external quantum efficiency (EQE) spectra were characterized using a CompactStat instrument (Ivium Technologies; Eindhoven, Netherlands) comprised of a power source (Abet Technologies 150 W xenon lamp, 13014) and a monochromator (DongWoo Opteron, MonoRa500i). The system was calibrated with the same photodiode detector for the solar simulator. Space-charge limited current (SCLC) measurement was performed using a Keithley 2400 sourcemeter. The scan range of the voltage was 0 V - 5 V and the measurement was conducted in a dark condition. The electrochemical impedance spectroscopy (EIS) measurements were conducted by applying 0.8 V in the dark, with a frequency range from 1 kHz to 1 Hz. External quantum efficiency of electroluminescence (EQE_EL_) was measured using a BEL-300 spectrometer from Bunkoukeiki. A DC voltage and current source/monitor (Advantest, R-6243) was used to adjust the applied voltage. All measurements were performed in air on glass-encapsulated samples.

**Rau analysis**

According to Rau et al., the *V*_OC_ can be estimated using the following equation (S1):

$V_{oc}=\frac{K_{B}T}{q}\ln\frac{J_{\mathrm{SC}}}{J_{0}}$	(S1),

which can be further expanded as equation (S2)

=$\frac{K_{B}T}{q}ln \frac{J_{\mathrm{SC}}^{\mathrm{SQ}}}{J_{0}^{\mathrm{SQ}}}+\frac{K_{B}T}{q}\ln\frac{J_{SC}}{J_{\mathrm{SC}}^{SQ}}+\frac{K_{B}T}{q}\ln\frac{J_{0}^{\mathrm{SQ}}}{J_{0}^{\mathrm{rad}}}+\frac{K_{B}T}{q}\ln\frac{J_{0}^{\mathrm{rad}}}{J_{0}}$,

$=$ $V_{\mathrm{OC}}^{SQ}-{\Delta V}_{\mathrm{SC}}-{\Delta V}_{\mathrm{rad}}-{\Delta V}_{\mathrm{nr}},$

$=\frac{E_{g}^{\mathrm{PV}}}{q}{-\Delta V}_{\mathrm{SQ}} -{\Delta V}_{\mathrm{SC}}-{\Delta V}_{\mathrm{rad}}-{\Delta V}_{\mathrm{nr}}$	(S2)

where ${\Delta V}_{SQ}= \frac{E_{g}^{\mathrm{PV}}}{q-V_{\mathrm{OC}}^{\mathrm{SQ}}}$ and the SQ represents Shockely-Queisser Limit. Here, $J_{\mathrm{SC}}^{\mathrm{SQ}},J_{0}^{\mathrm{SQ}}, J_{\mathrm{SC}} and J_{0}^{\mathrm{rad}}$ are given by the following equation (S3):

$$J_{\mathrm{SC}}^{\mathrm{SQ}}=q\int_{E_{g}}^{\infty} \Phi_{AM 1.5G}\left( E \right)dE,$$

$$J_{0}^{\mathrm{SQ}}=q\int_{E_{g}}^{\infty} \Phi_{\mathrm{BB}}\left( E \right)dE,$$

$$J_{\mathrm{SC}}=q\int_{E_{g}}^{\infty} \mathrm{EQE}_{\mathrm{PV}}(E)\Phi_{AM 1.5G}\left( E \right)dE,$$

$J_{0}^{\mathrm{rad}}=q\int_{E_{g}}^{\infty} {\mathrm{EQE}_{\mathrm{PV}}(E)\Phi}_{\mathrm{BB}}\left( E \right)dE,$	(S3)

where Φ_AM 1.5G_ and Φ_BB_ correspond to the AM1.5G solar spectrum and blackbody spectrum at *T* = 300 K of the solar cell, respectively. In the SQ framework, the fundamental voltage loss (${\Delta V}_{\mathrm{SQ}})$ is associated with radiative charge recombination and represents the theoretical thermodynamic limit for photovoltaic devices [3, 4]. However, additional voltage loss components, denoted as ∆*V*_SC_, ∆*V*_rad_, and ∆*V*_nr_, further influence device performance and are characterized as follows.

The ∆*V*_SC_ occurs when the *J*_SC_ falls below the theoretical maximum current density ($J_{\mathrm{SC}}^{\mathrm{SQ}}$), which assumes perfect (100%) conversion of incident photons with energies above $E_{g}^{\mathrm{PV}}$ into photocurrent. This loss is typically negligible. The ∆*V*_rad_ arises from radiative recombination pathways involving sub-bandgap photon, including contributions from the exponential absorption tails extending below the $E_{g}^{\mathrm{PV}}$. Finally, the ∆*V*_nr_ results from nonradiative recombination and is determined by the difference between the measured *V*_OC_ and the theoretical radiative-limit voltage ($V_{\mathrm{oc}}^{\mathrm{rad}}$).

**Computational details**

*A. Density functional theory (DFT) calculations*

All DFT calculations were conducted using DMol^3^ program [5, 6]. Generalized gradient approximation with Perdew-Burke-Ernzerhof functional was used to calculate the exchange-correlation energy [7]. Spin-polarized calculations were employed and Tkatchenko-Scheffler method was used for dispersion corrections [8] with the basis set of double numerical plus polarization (version 4.4). For the calculation of the electrostatic potential isosurface, Becke’s three-parameter hybrid exchange functional with Lee–Yang–Parr correlation functional was used for the exchange-correlation energy [9, 10]. For the calculation of molecular systems, the self-consistent field tolerance was set to 1 × 10^−6^ Ha. The geometry optimizations were performed with the convergence criteria of 1 × 10^−5^ Ha for energy, 0.002 Ha/Å for force, 0.005 Å for displacement. The core electrons were treated using the all electron relativistic method with the distance of orbital cutoff was set to 6.1 Å. For the calculation of periodic systems, the self-consistent field tolerance was set to 1 × 10^−5^ Ha. The geometry optimizations were performed with the convergence criteria of 2 × 10^−5^ Ha for energy, 0.004 Ha/Å for force, 0.005 Å for displacement. The core electrons were treated using the all electron method with the distance of orbital cutoff was set to 5.1 Å. For the periodic systems, 4 × 4 × 1 *k*-points were employed with a Monkhorst-Pack grid [11]. Furthermore, the unit cell of *α*-FAPbI_3_, which was optimized with 8 × 8 × 8 *k*-points.

The (100) surface of perovskite was selected for our DFT calculations, which was the dominant surface of *α*-FAPbI_3_ perovskite structure. FAI-terminated surface with FAI vacancy and I vacancy was considered to calculate the binding energy, defect formation energy, and O_2_ adsorption energy. Based on our experimental composition (*i.e.*, BA_0.02_(FA_0.83_Cs_0.17_)_0.98_Sn_0.5_Pb_0.5_I_3_), we constructed our model systems.

The binding energies between A-site (FA or Cs) cation and BX_3_ (SnI_3_ or PbI_3_) anion were calculated by following equation (S4)

Binding *E* = $\text{E}_{\text{total}}$ $-$ ($\text{E}_{\text{A}}$ + $\text{E}_{\text{BX}_{\text{3}}}$)	(S4)

where $\text{E}_{\text{total}}$, $\text{E}_{\text{A}}$, and $\text{E}_{\text{BX}_{\text{3}}}$ are the total energy of system, the energy of A-site cation, and the energy of BX_3_ anion, respectively.

The binding energies between the SnI_2_ or PbI_2_ precursors and SA molecule were calculated by following equation (S5)

Binding *E* = $\text{E}_{\text{total}}$ $-$ ($\text{E}_{\text{SnI}_{\text{2}}\text{ or}\text{ PbI}_{\text{2}}}$ + $\text{E}_{\text{SA}}$)	(S5)

where $\text{E}_{\text{total}}$, $\text{E}_{\text{SnI}_{\text{2}}\text{ or}\text{ PbI}_{\text{2}}}$, and $\text{E}_{\text{SA}}$ are the total energy of system, the energy of SnI_2_ or PbI_2_ molecule, and the energy of SA molecule, respectively.

The SA binding energies were calculated by following equation (S6)

Binding *E* = $\text{E}_{\text{total}}$ $-$ ($\text{E}_{\text{SA}}$ + $\text{E}_{\text{w/o SA}}$)	(S6)

where $\text{E}_{\text{total}}$, $\text{E}_{\text{SA}}$, and $\text{E}_{\text{w/o SA}}$ are the total energy of system, the energy of SA molecule, and without SA molecule in total system, respectively.

The defect formation energies were calculated by following equation (S7)

Defect formation *E* = ($\text{E}_{\text{I}\text{ vacancy}}$ + $\text{E}_{\text{I}\text{ }\text{atom}}$) $-$ $\text{E}_{\text{total}}$	(S7)

$\text{E}_{\text{I}\text{ vacancy}}$, $\text{E}_{\text{I}\text{ atom}}$ , and $\text{E}_{\text{total}}$ are the energy of the iodine vacancy system, the energy of iodine, and total energy of the system, respectively.

The O_2_ adsorption energies were calculated by following equation (S8)

Adsorption *E* = $\text{E}_{\text{total}}-\text{(}\text{E}_{\text{O}_{\text{2}}}+ \text{E}_{\text{w/o O}_{\text{2}}})$	(S8)

where $\text{E}_{\text{total}}$, $\text{E}_{\text{O}_{\text{2}}}$, and $\text{E}_{\text{w/o O}_{\text{2}}}$ are the total energy of system, the energy of O_2_ molecule, and without O_2_ molecule in total system, respectively.

**Figure S1.** Optimized structures and electrostatic potential isosurfaces of SnI_2_, PbI_2_, CsI, FAI, DMSO, and SA molecules. The isovalue is 0.03 *e*/Å^3^.

**Figure S2.** Optimized structures of (a) precursors, (b) precursors with DMSO molecule, and (c) precursors with DMSO and SA molecules. Note that precursors indicate that FA·SnI_3_, Cs·SnI_3_, FA·PbI_3_, and Cs·PbI_3_.

**Figure S3.** (a-d) Charge analysis of I atoms in precursors, precursors with DMSO molecule, and precursors with DMSO and SA molecules. (a) FA∙SnI_3_, (b) Cs∙SnI_3_, (c) FA∙PbI_3_, and (d) Cs∙PbI_3_ systems. (e) Ratio of increase in the negative charge of I atoms induced by the addition of DMSO and SA molecules to the precursor system.

**Figure S4.** Various model systems and optimized structures of (a-b) SA·SnI_2_, (c-d) SA·PbI_2_, and (e) binding energies of SA·SnI_2_ and SA·PbI_2_. Note that (a) and (c) indicate that model systems. (b) and (d) indicate the optimized structures by DFT calculations.

**Figure S5.** Molecular structure and simulated 1H NMR pattern of Sinapic acid (SA).

**Figure S6.** Photographs of the temporal stability for control and target perovskite solutions for 24 hours after air exposure.

**Figure S7.** Thermogravimetric analysis (TGA) of weight loss of pristine perovskite (PRV) without sinapic acid (SA), PRV with SA, and sinapic acid powders.

**Figure S8.** The ToF-SIMS depth profile for the control (without SA) and target (with SA) samples with ITO/HTL/perovskite films.

**Figure S9.** (a) XRD patterns for the control (without SA) and target (with SA) perovskite films. (b) FWHM values (left axis) and crystalline sizes (right axis) were calculated from the (100) lattice plane.

**Figure S10.** XRD patterns of perovskite films prepared by before annealing, 60 °C for 1 min, 100 °C for 2 min, 100 °C for 5 min, and 100 °C for 10 min.

**Figure S11.** FE-SEM morphological images of control perovskite films for before annealing, 60 °C for 1 min, 100 °C for 2 min, 100 °C for 5 min, and 100 °C for 10 min conditions and ImageJ-based statistical analysis on 100 grains from the FE-SEM images of the final films annealed at 100 °C for 10 min.

**Figure S12.** FE-SEM morphological images of target perovskite films for before annealing, 60 °C for 1 min, 100 °C for 2 min, 100 °C for 5 min, and 100 °C for 10 min conditions and ImageJ-based statistical analysis on 100 grains from the FE-SEM images of the final films annealed at 100 °C for 10 min.

**Figure S13.** (a) Model systems of FAI termination on perovskite surface with FAI vacancy defect. Note that the surface consists of five layers and 2 × 2 supercell along the *x*- and *y*- axes. The vacuum spacing is over 20 Å and two layers from the bottom are constrained. (b) Optimized structures and binding energies of BAI and FAI on the perovskite surface. Note that BAI molecule shows more thermodynamically favorable passivation than FAI molecule.

**Figure S14.** Optimized structures and total energies of BAI passivated perovskite surface according to Cs doping site. Note that the surface consists of five layers and 2 × 2 supercell along the *x*- and *y*- axes. The vacuum spacing is over 20 Å and two layers from the bottom are constrained.

**Figure S15.** (a) Model systems of FAI termination on perovskite surface with I vacancy defect. (b) Optimized structures of SA molecule on the perfect and I vacancy systems. Note that the surface consists of five layers and 2 × 2 supercell along the *x*- and *y*- axes. The vacuum spacing is over 20 Å and two layers from the bottom are constrained.

**Figure S16.** Optimized structures of w/o BA, control, and target systems with I vacancy defect*.*

**Figure S17.** Absorption spectra of the MA-free Sn-Pb perovskite films without and with sinapic acid. The inset is the tauc plots for the control and target perovskite films.

**Figure S18.** Plots of ln (*α*) versus *hν* to extract the Urbach energy (*E*_u_).

**Table S1.** The calculated PLQY values for the control and target perovskite films.

| Samples # | Control | Target |
| --- | --- | --- |
| 1 | $4.5 \times{10}^{-4}$ | $1.3 \times{10}^{-3}$ |
| 2 | $2.0 \times{10}^{-4}$ | $1.46 \times{10}^{-3}$ |
| 3 | $4.17 \times{10}^{-4}$ | $8.0 \times{10}^{-4}$ |
| 4 | $5.6 \times{10}^{-4}$ | $8.6 \times{10}^{-4}$ |

**Figure S19.** Steady-state photoluminescence (SS-PL) and time-resolved photoluminescence (TR-PL) spectra for glass/perovskite for control and target conditions.

**Table S2.** TR-PL decay obtained after fitting for glass/perovskite films for control and target shown in Figure S16.

|  | τ_1_ (ns) | τ_2_ (ns) | τ_avg_ (ns) |
| --- | --- | --- | --- |
| Control | 138.62 | 775.38 | 495.68 |
| Target | 31.12 | 728.67 | 600.80 |

**Figure S20.** SS-PL and TR-PL spectra for FTO glass/ANO (HTL)/perovskite for control and target conditions.

**Table S3.** TR-PL decay obtained after fitting for FTO glass/ANO (HTL)/perovskite films for control and target shown in Figure S17.

|  | τ_1_ (ns) | τ_2_ (ns) | τ_avg_ (ns) |
| --- | --- | --- | --- |
| Control | 12.46 | 343.57 | 20.48 |
| Target | 4.99 | 204.56 | 14.76 |

**Figure S21.** Time-dependent Absorption spectra of SnI_2_, SnI_2_+SA, SnI_2_+BA, SnI_2_+Tyrosol solutions under air exposure for 12 hours.

**Figure S22.** Photographs showing the color evolution of SnI_2_, SnI_2_+SA, SnI_2_+BA, SnI_2_+Tyrosol solutions in DMF+DMSO (4:1 v/v) during 60 min of air exposure.

**FigureS23.** The full spectra of XPS analysis for the control and target perovskite films.

**Table S4.** The ratio of Sn^2+^ and Sn^4+^ for Sn 3d of control and target perovskite films from XPS analysis in Figure 4b.

| Sample | Sn 3d_3/2_ | | Sn 3d_5/2_ | |
| --- | --- | --- | --- | --- |
|  | Sn^2+^ | Sn^4+^ | Sn^2+^ | Sn^4+^ |
| Control | 50.80 % | 49.19 % | 40.63 % | 59.36 % |
| Target | 74.70 % | 25.29 % | 77.97 % | 22.02 % |
| After 24 hours of aging (ambient conditions) | | | | |
| Control | 21.03 % | 78.96 % | 17.42 % | 82.57 % |
| Target | 40.89 % | 59.10 % | 43.37 % | 56.62 % |

**Figure S24.** XPS depth profile patterns for control (a) and target (b) perovskite films.

**Figure S25.** The fitted C 1s (a) and O 1s (b) XPS depth profile patterns at 0, 100, 200, and 300s for control perovskite films.

**Figure S26.** The fitted C 1s (a) and O 1s (b) XPS depth profile patterns at 0, 100, 200, and 300s for target perovskite films.

**Table S5**. The content ratios of each functionality in O 1s XPS depth profile patterns at 300s etching time for control and target perovskite films.

| O 1s XPS (300s etching time) | | | | | |
| --- | --- | --- | --- | --- | --- |
| Sample | Sn-O (Sn^2+^)/Pb-O  (%) | Sn-O_2_ (Sn^4+^)  (%) | C=O (%) | Chemisorbed O (%) | -OH/C-O (%) |
| Control | 13.0 | 67.87 |  | 19.07 |  |
| Target | 32.62 | 36.73 | 22.77 | 4.10 | 3.76 |

**Figure S27.** Electrostatic potential (ESP) charge extracted from the geometrically optimized sinapic acid (SA).

**Figure S28.** The fitted Sn 3d XPS depth profile patterns at 300s for control and target perovskite films.

**Table S6**. The ratios of each content in Sn 3d XPS depth profile patterns at 300s etching time for control and target perovskite films.

| Sn 3d XPS (300 s etching time) | | | | | | |
| --- | --- | --- | --- | --- | --- | --- |
| Sample | Sn 3d_5/2_ | | | Sn 3d_3/2_ | | |
|  | Sn^0^ | Sn^2+^ | Sn^4+^ | Sn^0^ | Sn^2+^ | Sn^4+^ |
| Control | 47.12 | 13.48 | 39.39 | 22.40 | 24.55 | 53.03 |
| Target | 43.38 | 35.16 | 21.45 | 24.77 | 46.73 | 28.48 |

**Figure S29.** Optimized structures and O_2_ adsorption energies for w/o BA, control, and target (I and II) systems with I vacancy defect.

**Figure S30.** UPS spectra of secondary electron cutoff and valence band of control and target perovskite films.

**Figure S31.** Energy band diagram for the MA-free Sn-Pb PSCs with control and target perovskites.

**Table S7.** Comparison of recently reported MA-free Sn-Pb PSCs.

| **Year** | **MA-free Sn-Pb perovskite/ additive strategy** | **HTL** | **Device area (cm^2^)** | **PCE (%)** | ***V*_OC_ (V)** | **Stability condition/ retention** | **Ref.** |
| --- | --- | --- | --- | --- | --- | --- | --- |
| 2024 | Rb/Dip cations + NaBH_4_ reducing agent + surface engineering (4FPEACl) for Cs_0.2_FA_0.8_ Sn–Pb perovskite | PEDOT:PSS | 0.0985 | 22.03 | 0.86 | 94% of the initial PCE after 700 h of light exposure with encapsulation | [12] |
| 2024 | MA-free BA_0.02_(Cs_0.17_FA_0.83_^)^_0.98_ Sn–Pb PSC with GeI_2_/GeO_x_ passivation (our previous work) | Al-NiO_x_ nanoparticle | 0.04 | 23.34 | 0.87 | Encapsulated: >95% after 4320 h; unencapsulated: 88% retained  Encapsulated device: T_85_ after 250 h under MPPT in air  Encapsulated device: 77% after 550 h at 55 ℃ in air | [1] |
| 2024 | Cs_0.2_FA_0.8_ Sn–Pb perovskite/ guanidine thiocyanate (GuaSCN) and aminoacetamide hydrochloride (AHC) | PEDOT:PSS | 0.093 | 22.14 | 0.861 | Unencapsulated device: 93% of their initial PCE after 1620 h in a N_2_ atmosphere at RT  Unencapsulated device: 89% after 240 h at 85 ℃ in N_2_  Encapsulated device: 91% after 550 h in air | [13] |
| 2025 | RbAC additive; MA-free FA_0.75_Cs_0.25_ Sn-Pb perovskite | PEDOT:PSS | 0.058 | 23.02 | 0.897 | Unencapsulated devices: 80% after 850 h light illumination in N_2_  60% after 120 h at 85 ℃ in N_2_ | [14] |
| 2025 | 1H-benzotriazol-1-yloxytris (dimethylamino) [phosphonium](https://www.sciencedirect.com/topics/chemistry/phosphonium) hexafluorophosphate (BOP) additive; MA-free FA-based Sn-Pb perovskite | HTL-free | 0.0975 | 22.23 | 0.85 | Almost independent after 6400 h in N_2_  86.96% after 200 h under MPPT in N_2_  80% after at 55 °C for 485 hours in N_2_ | [15] |
| 2025 | thiosemicarbazide hydrochloride (TH); Blade-coated all-FA Sn-Pb perovskite | PEDOT:PSS | 0.09 | 20.16 | 0.856 | Unencapsulated device: 80.5 % after 1000 h in N_2_  Unencapsulated device: 80 %  after 600 h in air  80 % after 50 hours at 85 °C in N_2_ | [16] |
| 2026 | multi-Lewis-base modulator (Gly, Glu, or Sc) additives, solvent-extraction channels (SSC); MA-free FA_0.85_Cs_0.15_-based Sn-Pb perovskite | PEDOT:PSS | 0.049 | 22.7 | 0.871 | Unencapsulated device: 80% after 800 h at 85 °C in N_2_ | [17] |
| 2026 | Hydrazinium dichloride additive; blade-coated all-FA Pb–Sn perovskite | PEDOT:PSS | 0.09 | 21.81 | 0.850 | Unencapsulated device: 80% after 190 hours  >85% after 1080 hours of continuous illumination | [18] |
| 2026 | This work | Al-NiO_x_ nanoparticle | 0.04, 1 | 23.2, 19.53 | 0.893, 0.890 | Unencapsulated device: >91% after 5200 h storage  Encapsulated device: 85% after 5200 h in air  Encapsulated device: 84% after 300 h under MPPT in air  Encapsulated device: 80% after 900 h at 50 °C in air |  |

**Figure S32.** *J*–*V* curves for the corresponding MA-free Sn-Pb perovskite solar cells with GA (gallic acid) and CA (caffeic acid).

**Figure S33.** EQE spectra and corresponding integrated *J*_sc_ curves of control and target cells.

**Figure S34.** Steady-state photocurrents for the PSCs without and with SA molecule for 600 s at the maximum power point.

**Figure S35.** *J–V* curves (forward and reverse scans) of the PSCs with the control and target conditions.

**Figure S36.** *J–V* curve of the target PSC with the highest fill factor.

**Figure S37.** Box charts illustrating the photovoltaic parameters of *J*_SC_, FF, and PCE for 20 devices.

**Table S8.** Photovoltaic performance parameters of the control devices under AM 1.5 illustration (100 mW/cm^2^) measured in the air (RT, RH: 25-40%).

| Control # | *V*_OC_ (V) | *J*_SC_ (mA/cm^2^) | FF (%) | PCE (%) |
| --- | --- | --- | --- | --- |
| 1 | 0.834 | 32.21 | 70.6 | 19.0 |
| 2 | 0.829 | 32.00 | 71.8 | 19.1 |
| 3 | 0.830 | 31.99 | 72.8 | 19.3 |
| 4 | 0.798 | 32.50 | 70.7 | 18.3 |
| 5 | 0.809 | 32.38 | 72.7 | 19.0 |
| 6 | 0.807 | 32.26 | 72.3 | 18.8 |
| 7 | 0.823 | 32.98 | 72.9 | 19.8 |
| 8 | 0.824 | 32.95 | 72.7 | 19.8 |
| 9 | 0.827 | 33.43 | 72.2 | 20.0 |
| 10 | 0.790 | 33.25 | 71.8 | 18.8 |
| 11 | 0.827 | 33.23 | 75.2 | 20.7 |
| 12 | 0.820 | 33.65 | 71.0 | 19.6 |
| 13 | 0.821 | 33.58 | 70.7 | 19.5 |
| 14 | 0.823 | 33.54 | 71.6 | 19.8 |
| 15 | 0.820 | 32.96 | 75.1 | 20.3 |
| 16 | 0.826 | 33.50 | 70.9 | 19.6 |
| 17 | 0.833 | 33.18 | 66.3 | 18.3 |
| 18 | 0.826 | 33.34 | 67.5 | 18.6 |
| 19 | 0.821 | 33.35 | 67.7 | 18.5 |
| 20 | 0.817 | 33.25 | 67.5 | 18.4 |
| Average | 0.820 ± 0.011 | 32.98 ± 0.55 | 71.2 ± 2.39 | 19.3 ± 0.69 |

**Table S9.** Photovoltaic performance parameters of the target devices under AM 1.5 illustration (100 mW/cm^2^) measured in the air (RT, RH: 25-40%).

| Target # | *V*_OC_ (V) | *J*_SC_ (mA/cm^2^) | FF (%) | PCE (%) |
| --- | --- | --- | --- | --- |
| 1 | 0.886 | 33.67 | 75.6 | 22.5 |
| 2 | 0.888 | 33.29 | 76.3 | 22.6 |
| 3 | 0.889 | 32.63 | 77.2 | 22.4 |
| 4 | 0.890 | 33.22 | 75.4 | 22.3 |
| 5 | 0.876 | 33.36 | 77.7 | 22.7 |
| 6 | 0.870 | 33.42 | 78.4 | 22.7 |
| 7 | 0.888 | 32.86 | 77.4 | 22.6 |
| 8 | 0.881 | 33.41 | 76.1 | 22.4 |
| 9 | 0.888 | 32.16 | 80.3 | 22.9 |
| 10 | 0.885 | 32.14 | 81.0 | 23.0 |
| 11 | 0.892 | 33.16 | 77.2 | 22.8 |
| 12 | 0.893 | 33.74 | 75.1 | 22.6 |
| 13 | 0.889 | 33.74 | 76.1 | 22.8 |
| 14 | 0.889 | 33.48 | 75.6 | 22.6 |
| 15 | 0.890 | 33.21 | 77.5 | 22.9 |
| 16 | 0.893 | 33.42 | 77.7 | 23.2 |
| 17 | 0.888 | 33.29 | 77.5 | 22.9 |
| 18 | 0.882 | 32.95 | 77.3 | 22.5 |
| 19 | 0.870 | 33.68 | 77.6 | 22.6 |
| 20 | 0.870 | 33.47 | 78.1 | 22.7 |
| Average | 0.885 ± 0.007 | 33.22 ± 0.46 | 77.3 ± 1.51 | 22.7 ± 0.22 |

**Figure S38.** *J*–*V* curves of the best-performing 1cm^2^ large-area control and target devices.

|  | *V*_OC_ (V) | *J*_SC_ (mA/cm^2^) | FF (%) | PCE(%) |
| --- | --- | --- | --- | --- |
| Control | 0.817 | 30.80 | 64.52 | 16.23 |
| Target | 0.890 | 31.19 | 70.35 | 19.53 |

**Table S10.** Photovoltaic parameters of the best-performing 1 cm^2^ large-area devices for control and target under AM 1.5G illumination.

| Device | *V*_OC_ (V) | *J*_SC_ (mA/cm^2^) | FF (%) | PCE(%) |
| --- | --- | --- | --- | --- |
| Control-1 | 0.817 | 30.80 | 64.52 | 16.23 |
| Control-2 | 0.808 | 30.94 | 62.14 | 15.53 |
| Control-3 | 0.815 | 30.58 | 63.20 | 15.75 |
| Control-4 | 0.810 | 30.63 | 63.46 | 15.74 |
| Control-5 | 0.813 | 30.82 | 61.68 | 15.45 |
| Control average | 0.813 ± 0.004 | 30.75 ± 0.15 | 63.00 ± 1.12 | 15.74 ± 0.30 |
| Target-1 | 0.890 | 31.19 | 70.35 | 19.53 |
| Target-2 | 0.888 | 31.08 | 70.20 | 19.37 |
| Target-3 | 0.885 | 31.22 | 70.18 | 19.39 |
| Target-4 | 0.887 | 31.04 | 70.68 | 19.46 |
| Target-5 | 0.882 | 31.15 | 70.54 | 19.38 |
| Target average | 0.886 ± 0.003 | 31.14 ± 0.08 | 70.39 ± 0.22 | 19.43 ± 0.07 |

**Table S11.** Photovoltaic parameters of the 1 cm^2^ large-area devices (5 samples) for control and target under AM 1.5G illumination.

**Figure S39.** EQE combined with a semilogarithmic plot of the EL spectra of the control and target devices.

|  | *E*_g_ / eV | *qV*_OC_ / eV | *q∆V*_SQ_/ eV | *q∆V*_SC_ / eV | *q∆V*_rad_/ eV | *q∆V*_nr_/ eV |
| --- | --- | --- | --- | --- | --- | --- |
| **Control** | 1.25 | 0.83 | 0.243 | 0.018 | 0.013 | **0.171** |
| **Target** | 1.25 | 0.89 | 0.243 | 0.019 | 0.015 | **0.112** |

**Table S12.** The voltage loss parameters estimated by Rau analysis from Figure 4h and 4i.

**Table S13.** The FF values for PSCs with and without the SA molecule by evaluated based on experimental data using Equation (4–6).

|  | FF^exp^ | FF^cal^ | FF^exp^/FF^cal^ |
| --- | --- | --- | --- |
| Control | 0.752 | 0.802 | 0.937 |
| Target | 0.777 | 0.845 | 0.919 |

**Table S14.** The relatively contributions of these parameters to FF enhancement from control to target conditions.

|  | *n*_id_ | *V*_OC_ | *R*_s_ | *R*_sh_ |
| --- | --- | --- | --- | --- |
| $\mathrm{FF}_{x}^{\text{cal}}$-$\mathrm{FF}_{\mathrm{con}}^{\text{cal}}$ | 0.017 | 0.007 | 0.005 | 0.012 |
| % | 42 | 18 | 11 | 29 |

X indicates the variable that was changed from its baseline value in the corresponding experimental condition of target. All other parameters were kept constant to evaluate the isolated impact of that single variable.

**Figure S40.** Long-term stability of the unencapsulated 1 cm^2^ large-area devices corresponding conditions in ambient air.

**Figure S41.** XRD patterns of the control and target devices for initial and aging (after 30 days) conditions. Insets are photographs of initial and aging devices.

**References**

[1]	S. Lee, J. Ryu, D.-G. Lee, et al., "Unprecedented inorganic HTL-based MA-free Sn–Pb perovskite photovoltaics with an efficiency over 23%" *Energy & Environmental Science* 17 (2024): 8140-8150, <https://doi.org/10.1039/D4EE03579H>

[2]	F. J. Berger, I. Poli, E. Aktas, et al., "How halide alloying influences the optoelectronic quality in tin-halide perovskite solar absorbers" *ACS Energy Letters* 8 (2023): 3876-3882, <https://doi.org/10.1021/acsenergylett.3c01241>

[3]	W. Shockley, H. J. Queisser, "Detailed Balance Limit of Efficiency of p‐n Junction Solar Cells" *Journal of Applied Physics* 32 (1961): 510-519, <https://doi.org/10.1063/1.1736034>

[4]	T. Kirchartz, U. Rau, M. Kurth, et al., "Comparative study of electroluminescence from Cu (In, Ga) Se2 and Si solar cells" *Thin Solid Films* 515 (2007): 6238-6242, <https://doi.org/10.1016/j.tsf.2006.12.105>

[5]	B. Delley, "An all‐electron numerical method for solving the local density functional for polyatomic molecules" *The Journal of chemical physics* 92 (1990): 508-517, <https://doi.org/10.1063/1.458452>

[6]	B. Delley, "From molecules to solids with the DMol^3^ approach" *The Journal of chemical physics* 113 (2000): 7756-7764, <https://doi.org/10.1063/1.1316015>

[7]	J. P. Perdew, K. Burke, M. Ernzerhof, "Generalized gradient approximation made simple" *Physical review letters* 77 (1996): 3865, <https://doi.org/10.1103/PhysRevLett.77.3865>

[8]	A. Tkatchenko, M. Scheffler, "Accurate Molecular Van Der Waals Interactions from Ground-State Electron Density and Free-Atom Reference Data" *Physical review letters* 102 (2009): 073005, <https://doi.org/10.1103/PhysRevLett.102.073005>

[9]	A. D. Becke, "Density‐functional thermochemistry. III. The role of exact exchange" *The Journal of chemical physics* 98 (1993): 5648-5652, <https://doi.org/10.1063/1.464913>

[10]	P. J. Stephens, F. J. Devlin, C. F. Chabalowski, et al., "Ab initio calculation of vibrational absorption and circular dichroism spectra using density functional force fields" *The Journal of physical chemistry* 98 (1994): 11623-11627, <https://doi.org/10.1021/j100096a001>

[11]	H. J. Monkhorst, J. D. Pack, "Special points for Brillouin-zone integrations" *Physical review B* 13 (1976): 5188, <https://doi.org/10.1103/PhysRevB.16.1746>

[12]	S.-H. Turren-Cruz, J. Pascual, S. Hu, et al., "Multicomponent approach for stable methylammonium-free tin–lead perovskite solar cells" *ACS Energy Letters* 9 (2024): 432-441, <https://doi.org/10.1021/acsenergylett.3c02426>

[13]	J. Wang, Y. Pan, Z. Zhou, et al., "Bimolecular Crystallization Modulation Boosts the Efficiency and Stability of Methylammonium‐Free Tin–Lead Perovskite and All‐Perovskite Tandem Solar Cells" *Advanced Energy Materials* 14 (2024): 2402171, <https://doi.org/10.1002/aenm.202402171>

[14]	X. Liao, X. Jia, W. Li, et al., "Methylammonium-free, high-efficiency, and stable all-perovskite tandem solar cells enabled by multifunctional rubidium acetate" *Nature communications* 16 (2025): 1164, <https://doi.org/10.1038/s41467-025-56549-8>

[15]	J. You, Q. Gao, J. Wang, et al., "Crystallization control and interface passivation for efficient hole transport layer-free and methylammonium-free low-bandgap tin-lead perovskite solar cells" *Nano Energy* 142 (2025): 111209, <https://doi.org/10.1016/j.nanoen.2025.111209>

[16]	Y. Liu, H. Li, J. Wei, et al., "Retarding crystallization for high-quality printable All-FA-based Sn-Pb perovskite thin-films and all-perovskite tandem photovoltaic devices" *Nano Energy* 141 (2025): 111093, <https://doi.org/10.1016/j.nanoen.2025.111093>

[17]	Y. Guo, M. Yin, H. Gao, et al., "Homogeneous crystallization via sustained solvent-extraction channels for methylammonium-free all-perovskite tandem solar cells" *Nature communications* (2026): <https://doi.org/10.1038/s41467-025-68213-2>

[18]	H. Li, W. Feng, J. Wei, et al., "Synchronizing crystallization enables thermally stable all-FA Pb–Sn perovskites for printable MA-free all-perovskite tandem solar cells" *Energy & Environmental Science* 19 (2026): 1296-1305, <https://doi.org/10.1039/D5EE04529K>
